# Protein intake and cancer: an umbrella review of systematic reviews for the evidence-based guideline of the German Nutrition Society

**DOI:** 10.1007/s00394-024-03380-4

**Published:** 2024-04-21

**Authors:** Tilman Kühn, Nicole Kalotai, Anna M. Amini, Julia Haardt, Andreas Lehmann, Annemarie Schmidt, Anette E. Buyken, Sarah Egert, Sabine Ellinger, Anja Kroke, Stefan Lorkowski, Sandrine Louis, Matthias B. Schulze, Lukas Schwingshackl, Roswitha Siener, Gabriele I. Stangl, Bernhard Watzl, Armin Zittermann, Katharina Nimptsch, on behalf of the German Nutrition Society

**Affiliations:** 1 The Institute for Global Food Security, Queen’s University Belfast, 19 Chlorine Gardens, Belfast, Northern Ireland BT9 5DL UK; 2 Heidelberg Institute of Global Health (HIGH), Faculty of Medicine and University Hospital, Heidelberg, Germany; 3 Department of Nutritional Sciences, University of Vienna, Vienna, Austria; 4 Center for Public Health, Medical University of Vienna, Vienna, Austria; 5 German Nutrition Society, Bonn, Germany; 6 Institute of Nutrition, Consumption and Health, Faculty of Natural Sciences, Paderborn University, Paderborn, Germany; 7 Institute of Nutritional and Food Science, Nutritional Physiology, University of Bonn, Bonn, Germany; 8 Institute of Nutritional and Food Science, Human Nutrition, University of Bonn, Bonn, Germany; 9 Department of Nutritional, Food and Consumer Sciences, Fulda University of Applied Sciences, Fulda, Germany; 10 Institute of Nutritional Sciences, Friedrich Schiller University Jena, Jena, Germany; 11 Competence Cluster for Nutrition and Cardiovascular Health (nutriCARD) Halle-Jena-Leipzig, Jena, Germany; 12 Department of Physiology and Biochemistry of Nutrition, Max Rubner-Institut, Karlsruhe, Germany; 13 Department of Molecular Epidemiology, German Institute of Human Nutrition Potsdam-Rehbruecke, Nuthetal, Germany; 14 Institute of Nutritional Science, University of Potsdam, Potsdam, Germany; 15 Institute for Evidence in Medicine, Medical Center, University of Freiburg, Freiburg, Germany; 16 Department of Urology, University Stone Center, University Hospital Bonn, Bonn, Germany; 17 Institute of Agricultural and Nutritional Sciences, Martin Luther University Halle-Wittenberg, Halle (Saale), Germany; 18 Clinic for Thoracic and Cardiovascular Surgery, Herz- und Diabeteszentrum Nordrhein-Westfalen, Ruhr University Bochum, Bad Oeynhausen, Germany; 19 Molecular Epidemiology Research Group, Max Delbrück Center for Molecular Medicine (MDC) in the Helmholtz Association, Robert-Rössle-Straße 10, 13125 Berlin, Germany

**Keywords:** Protein, Umbrella review, Evidence-based guideline, Cancer

## Abstract

**Purpose:**

It has been proposed that a higher habitual protein intake may increase cancer risk, possibly via upregulated insulin-like growth factor signalling. Since a systematic evaluation of human studies on protein intake and cancer risk based on a standardised assessment of systematic reviews (SRs) is lacking, we carried out an umbrella review of SRs on protein intake in relation to risks of different types of cancer.

**Methods:**

Following a pre-specified protocol (PROSPERO: CRD42018082395), we retrieved SRs on protein intake and cancer risk published before January 22th 2024, and assessed the methodological quality and outcome-specific certainty of the evidence using a modified version of AMSTAR 2 and NutriGrade, respectively. The overall certainty of evidence was rated according to predefined criteria.

**Results:**

Ten SRs were identified, of which eight included meta-analyses. Higher total protein intake was not associated with risks of breast, prostate, colorectal, ovarian, or pancreatic cancer incidence. The methodological quality of the included SRs ranged from *critically low* (kidney cancer), *low* (pancreatic, ovarian and prostate cancer) and *moderate* (breast and prostate cancer) to *high* (colorectal cancer). The outcome-specific certainty of the evidence underlying the reported findings on protein intake and cancer risk ranged from *very low* (pancreatic, ovarian and prostate cancer) to *low* (colorectal, ovarian, prostate, and breast cancer). Animal and plant protein intakes were not associated with cancer risks either at a *low* (breast and prostate cancer) or *very low* (pancreatic and prostate cancer) outcome-specific certainty of the evidence. Overall, the evidence for the lack of an association between protein intake and (i) colorectal cancer risk and (ii) breast cancer risk was rated as *possible*. By contrast, the evidence underlying the other reported results was rated as *insufficient*.

**Conclusion:**

The present findings suggest that higher total protein intake may not be associated with the risk of colorectal and breast cancer, while conclusions on protein intake in relation to risks of other types of cancer are restricted due to *insufficient* evidence.

**Supplementary Information:**

The online version contains supplementary material available at 10.1007/s00394-024-03380-4.

## Introduction

The global incidence of cancer is on the increase. It is expected that cancer will become the most frequent cause of death in most countries worldwide during the twenty-first century [1, 2]. Despite advances in early detection and treatment, many cancer patients still have a lower life expectancy compared to persons without cancer. Moreover, the growing number of cancer survivors face major challenges with respect to tumour recurrence, comorbidities, psychosocial distress, and impairments to quality of life [3].

Around 40 percent of all cancers may be attributable to modifiable risk factors, and thus amenable to primary prevention [4–6]. Recent projections suggest that after smoking, which may explain 19.3% of all cancers [7], an unhealthy diet is the second most important cancer risk factor, with an attributable fraction of 7.8% due to low intakes of fruit, non-starchy vegetables, and fibre, and high intakes of salt, as well as red and processed meat [8]. The latter components of an unhealthy diet were derived from the World Cancer Research Fund’s (WCRF) expert report on diet and cancer, a landmark compilation of systematic reviews (SRs) on associations between dietary factors and risks of different cancer types.

While increased risks of cancer and particularly colorectal cancer among regular consumers of red and processed meat have been explained by higher intakes of heterocyclic amines, polycyclic aromatic hydrocarbons, N-nitroso compounds, or haem iron [9], it has also been suggested that higher protein intake of animal origin—which may result from a high meat consumption—may be a dietary cancer risk factor [10]. Interestingly, laboratory-based mechanistic studies indicate that protein restriction may protect against cancer due to decreased systemic insulin-like growth factor 1 (IGF-1) signalling and a subsequent downregulation of intracellular mTor activation [11, 12].

While SRs on protein intake and risks of certain cancers have been published [13–22], a standardised assessment of the epidemiological evidence is lacking. Thus, we carried out an umbrella review of SRs of human studies on protein intake (overall, as well as animal and plant protein) and cancer risk (overall and by cancer type) based on standardised approaches to evaluate the quality of published SRs and to grade the certainty of the evidence. Our study is part of a series of umbrella reviews on protein intake and health-related outcomes, carried out as the basis of a new Protein Guideline by the German Nutrition Society [23].

## Methods

We conducted an umbrella review (PROSPERO: CRD42018082395) following the methodological protocol published by Kroke et al. [23]. This protocol was developed as part of the evaluation of protein intake and various health-related outcomes and was also used for cancer. Two authors conducted all methodological steps independently. Any disagreements were resolved by discussion to achieve consensus.

### Literature search

The systematic literature search was conducted in PubMed, Embase and Cochrane Database of Systematic Reviews for SRs published between 1st July/2009 and 22th January/2024. The date of 1st July 2009 originates from the decision to cover a ten-year period, i.e. the initial database search was conducted on 11th July 2019, and the last update was made on 22th January 2024 to capture the current status of research. Because this time period refers to the publication of the SRs, older primary literature is also taken into account. The search strategy is presented in the Supplementary Materials (S1). In addition to the systematic literature search in the three databases, SRs proposed by members of the guideline expert panel on protein of the German Nutrition Society were also included in the literature selection process.

### Literature selection

Titles and/or abstracts of retrieved studies were screened according to pre-defined inclusion and exclusion criteria to identify potentially eligible studies [23]. The full-texts of potentially relevant publications were retrieved and assessed for eligibility. It was tolerated that some of the primary studies were incorporated more than once in different SRs. An overview of primary studies included in different SRs is shown in the Supplementary Materials (S2).

Publications were included if they met the following pre-specified criteria [23]: (i) SR evaluated the association between protein intake (total, animal-derived, plant-derived, from supplements) and cancer, (ii) population was the general adult population including older adults and recreational athletes, (iii) publication type was an SR with or without meta-analysis of prospective studies with human study participants, i. e. randomised controlled trials (RCTs), prospective cohort studies, case-cohort studies, or nested case–control studies. SRs also considering case–control studies were only included if prospective studies were predominant (> 50% of all studies), (iv) SR was written in English or German, and (v) SR was published between 07/2009 and 01/2024.

SRs of studies among children, adolescents, pregnant women, breastfeeding women, or top athletes were excluded**,** and so were individual studies, umbrella reviews, and SRs restricted to case–control studies, cross-sectional studies, or case studies. SRs of studies based on whole food approaches, in which protein intake was not specifically investigated, and studies on peptide and amino acid intakes were also excluded [23].

### Data extraction

The following data from each included SR were extracted into a standardised Excel table: the first author’s surname, year of publication, study type (e.g. SR with meta-analysis of RCTs), duration range of primary studies, study population, intervention/exposure(s), outcome(s), effect estimate(s) including 95% confidence intervals (CI), and heterogeneity estimate(s).

### Methodological quality of SRs

To assess the methodological quality of the retrieved SRs, a modified version of the AMSTAR 2 (A Measurement Tool to Assess Systematic Reviews 2) tool [24] was used (Supplementary Materials (S3)). The modifications and the rationale for such modifications are described in detail in our methodological protocol [23]. This version of AMSTAR 2 contains 14 items evaluating the methodological quality of the SR. SRs were rated on a scale from high quality to critically low quality according to the existence of critical and non-critical methodological weaknesses. SRs rated as „critically low” by AMSTAR 2 were excluded from the rating of the overall certainty of evidence.

### Outcome-specific certainty of evidence of SRs

The outcome-specific certainty of evidence of included SRs with and without meta-analysis was rated using the NutriGrade scoring tool [25] (Supplementary Materials (S4)). NutriGrade aims to assess the certainty of evidence of an association or effect between different dietary factors and outcomes, taking into account nutrition research-specific requirements not considered by other tools. This tool utilises a numerical scoring system and comprises seven items for SRs with meta-analysis of RCTs and eight items for SRs with meta-analysis of cohort studies. Based on the scoring system, four categories rate the potential outcome-specific certainty of evidence: high, moderate, low, and very low. The NutriGrade scoring tool was modified for the assessment of SRs without meta-analysis [23] (Supplementary Materials (S5)). We have adjusted the items related to meta-analyses: (i) precision: the confidence intervals were deleted, (ii) heterogeneity: this item was reduced to the question about consistency of the results, (iii) publication bias: this item was deleted, (iv) effect size: the RR/HR were deleted and (v) dose–response: this item was deleted. For SRs reporting more than one relevant outcome, each outcome was assessed separately. For SRs reporting more than one relevant exposure-outcome association, each exposure-outcome association was assessed separately.

### Approaches to assess overall certainty of evidence and deriving conclusions

To reach a conclusion regarding protein intake and cancer, we proceeded in three steps. First, we assessed the methodological quality of retrieved SRs. Second, we used a scoring tool to assess the certainty of evidence of an association or effect between protein intake and the incidence of various cancer sites. Third, we rated the overall certainty of evidence separately for each relevant exposure–outcome association considering all relevant SRs and according to the framework outlined in the methodological protocol [23] and in Table 1. Briefly, the overall rating ranges from convincing, probable, possible to insufficient. At first, we assessed whether there is at least one SR with or without meta-analysis of prospective studies. If more than one SR with or without meta-analysis was available, all (convincing) or the majority (probable, possible) of the results must be consistent. Biological plausibility must be given in any case (direct or inverse association). In the final step, the results of the AMSTAR 2 and NutriGrade ratings were considered. Depending on the level of evidence, the SRs must have achieved a certain rating in both tools. If no SR is identified, or if the majority of SRs reached a very low outcome-specific certainty of evidence and/or low methodological quality, the overall certainty of evidence was considered insufficient. For this publication, two authors (KN and TK) made suggestions for rating the overall certainty of evidence. This rating was double-checked by a staff member of the German Nutrition Society (NK) and thereafter reviewed by all co-authors. The final ratings of the overall certainty of evidence was approved by all authors.

**Table 1:** Grading the overall certainty of evidence according to methodological quality, outcome-specific certainty of evidence, biological plausibility and consistency of results, and definition of the overall certainty of evidence in a modified form according to the GRADE approach [23, 26]

Overall certainty of evidence	Underlying criteria	Definition/explanation
Convincing	• At least one SR with or without MA of prospective studies available • If more than one SR with or without MA are available: all overall results must be consistent.^1^ • In case of a positive or negative association, biological plausibility is given • All included SRs with or without MA must reach at least a “moderate” outcome-specific certainty of evidence^2^; in addition all included SRs must reach at least a methodological quality^3^ of “moderate”	There is high level of confidence that the true effect lies close to that of the estimate(s) of the effect
Probable	• At least one SR with or without MA of prospective studies available • If more than one SR with or without MA are available, the majority of overall results must be consistent.^1^ • In case of a positive or negative association, biological plausibility is given • The majority^4^ of included SRs with or without MA must have reached at least a “moderate” certainty of evidence^2^; in addition all included SRs must reach at least a methodological quality^3^ of “moderate”	There is moderate confidence in the effect estimate(s): The true effect is likely to be close to the estimate of the effect, but there is a possibility that it is substantially different
Possible	• At least one SR with or without MA of prospective studies available • If more than one SR with or without MA are available, the majority of overall results must be consistent.^1^ • In case of a positive or negative association, biological plausibility is given • The majority^4^ of included SRs with or without MA must reach at least a “low” certainty of evidence^2^; in addition the majority^4^ of all included SRs must reach at least a methodological quality^3^ of “moderate”	Confidence in the effect estimate(s) is limited: The true effect may be substantially different from the estimate of the effect
Insufficient	• No SR is available *OR* • The majority^4^ of included SRs with or without MA reach a “very low” certainty of evidence^2^; in addition the majority of all included SRs reach a methodological quality^3^ of “low”	There is very little confidence in the effect estimate (s): The true effect is likely to be substantially different from the estimate of effect

*MA* meta-analysis, *SR* systematic review

^1^Consistent = overall results of the SR have to be consistently either risk reducing or risk elevating or consistently showing no risk association

^2^Outcome-specific certainty of evidence refers to the NutriGrade rating

^3^Methodological quality refers the AMSTAR 2 rating**;** SRs graded as “critically low” by AMSTAR 2 are not considered

^4^Majority: > 50% of the included SRs

## Results

**Fig. 1:**
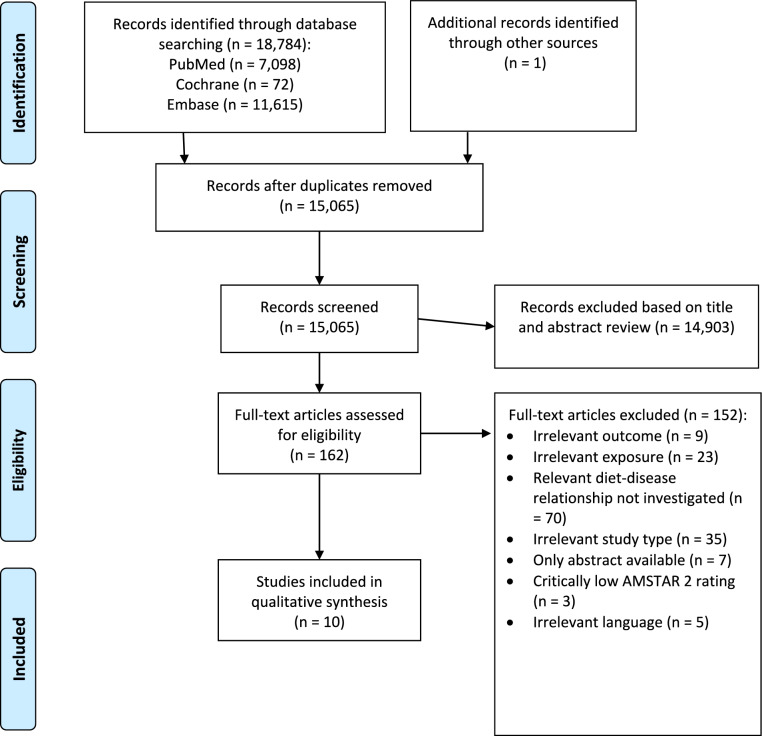
Flow diagram on systematic reviews included

In total, 18,785 records were initially identified by literature search. An additional reference is based on the guideline expert panel’s recommendation. This reference is an analysis of the WCRF that we included in the literature selection process. After the removal of duplicates, 15,066 records were screened based on title and abstract. We identified 163 potentially eligible records, and 10 were considered to be eligible with respect to inclusion and exclusion criteria [13–22]. The literature selection process is outlined in the flow diagram shown in Fig. 1. A list of excluded studies after full-text screening, including justifications for exclusion, is provided in Supplementary Materials (S6). Three SRs [27–29] were excluded from the evaluation because of a “critically low” AMSTAR 2 rating. The reason for exclusion was that Gathirua-Mwangi et al., Wu et al., and the WCRF report failed both to use a comprehensive literature search strategy and to provide an adequate risk of bias assessment. Additionally, the WCRF report failed to carry out an adequate investigation of publication bias.

### Characteristics of the included SRs

Table 2 shows the characteristics of the 10 included SRs. None considered RCTs according to the search criteria defined by the respective authors, seven were SRs of only cohort studies [13–15, 17, 18, 20, 21], two were SRs of cohort and case control studies [16, 19], and one was an SR of cohort and nested case–control studies [22]. Two out of the ten retrieved SRs did not include a meta-analysis [17, 22]. Dose–response analyses were conducted in one of the eight meta-analyses [14].

**Table 2:** Characteristics of the included systematic reviews

A) Systematic reviews with meta-analysis									
Author, year	Study type, study period	Study population	Exposition	Protein intake	Outcome	Effect estimates RR (95% CI)	Heterogeneity estimators	NutriGrade rating	AMSTAR 2 rating
Shin 2023 [13]	- Published before 12/2022 - Follow-up: 7.4 yr	- Only females - age NP	High *vs.* low protein intake	> 12.8 *vs.* ≤ 4.87 g/d Dietary assessment methods NP	Breast cancer				Moderate
	1 cohort study	*n* = 73,223 participants *n* = 592 cases	Soy protein			0.89 (0.67,1.17)^1^	*I*^2^ = 0%	NA	
Alzahrani 2022 [14]	- Published before 10/2021 - Follow-up: 3.5–18 yr	- Only males - Aged 26–92 yr	High *vs.* low protein intake	Protein intake NP Dietary assessment methods: selfreported FFQ, selfreported food record, recall interview	Prostate cancer				Moderate
	8 cohort studies	*n* = 214,276 participants *n* = 12,567 cases	Total protein	90.0–128.7 g/d *vs.* 38.0–82.0 g/d		0.99 (0.92, 1.07)^2^	*I*^2^ = 12.8% *P* = 0.33	Low: 4.9	
	7 cohort studies	*n* = 204,060 participants *n* = 7314 cases				Dose–response analysis: 0.99 (0.98, 1.01) per 20 g/d increase *P*_non-linearity_ = 0.51	*I*^2^ = 0% *P* = 0.43		
	6 cohort studies	*n* = 324,197 participants *n* = 26,207 cases	Animal protein	62.8–80 g/d *vs.* 8.5–47 g/d		0.99 (0.95, 1.04)	*I*^2^ = 0% *P* = 0.94	Low: 5.9	
	5 cohort studies	*n* = 313,981 participants *n* = cases				Dose–response analysis: 1.00 (0.98, 1.01) per 20 g/d increase *P*_non-linearity_ = 0.34	*I*^2^ = 25.5% *P* = 0.25		
	6 cohort studies	*n* = 329,082 participants *n* = 26,137 cases	Plant protein	27.9–47.0 g/d *vs.* 13.0–29.0 g/d		1.01 (0.96, 1.06)	I^2^ = 0% *P* = 0.63	Low: 4.9	
	4 cohort studies	*n* = 304,866 participants *n* = 20,704 cases				Dose–response analysis: 1.01 (0.98, 1.04) per 20 g/d increase *P*_non-linearity_ = 0.81	I^2^ = 0% *P* = 0.57		
	4 cohort studies	*n* = 167,489 participants *n* = 9,864 cases	Dairy protein	20.5–27.0 g/d *vs.* 6.3–10.0 g/d		1.08 (1.00, 1.16)	I^2^ = 38.1% *P* = 0.16	Moderate: 6.0	
	3 cohort studies	*n* = 157,273 participants *n* = 4611 cases				Dose–response analysis: 1.10 (1.02, 1.20) per 20 g/d increase *"Dairy protein intakes from 30 g/d to higher amounts were associated with a higher risk of prostate cancer, while this association for the intakes of* < *30 g/d was not significant."* *P*_non-linearity_ = 0.02	I^2^ = 42.5% *P* = 0.17		
Fan 2022 [15]	- Published before 09/2021 - Follow-up: 5.2–14 yr	- Both sexes - Aged 40–74 yr	High *vs.* low protein intake	Protein intake NP Dietary assessment methods: 77-item validated FFQ, 81-item validated FFQ, 45-item validated FFQ, 165-item validated FFQ	Overall cancer incidence				Moderate
	6 cohort studies	*n* = 349,158 participants *n* = 1,970 cases	Soy protein			0.95 (0.71, 1.28)^1^	*I*^2^ = 68.9% *P* = 0.007	Low: 4.3	
Zhang 2022 [16]	- Published before 10/2019 - Follow-up: NP	- Both sexes - Aged 34–89 yr	High *vs.* low protein intake	Protein intake NP Dietary assessment methods: FFQ, validated FFQ	Pancreatic cancer				Low
	2 cohort studies	*n* = 77,156 participants *n* = 217 cases	Total protein			0.98 (0.63, 1.54)^1^	*I*^2^ = 0% *P* = 0.758	Very low: 1.5	
	2 cohort studies and 1 case–control study	*n* = 78,134 participants *n* = 543 cases	Animal protein			1.37 (0.93, 2.01)^1^	*I*^2^ = 32.7% *P* = 0.226	Very low: 2.5	
	2 cohort studies and 1 case–control study	*n* = 78,134 participants *n* = 543 cases	Plant protein			0.78 (0.54, 1.14)^1^	*I*^2^ = 11.5% *P* = 0.323	Very low: 2.5	
Khodavandi 2020 [18]	- Published before 11/2019 - Follow-up: 8.1–16 yr	- Only females - Aged 25–84 yr	High *vs.* low protein intake	> 83.5 g/d *vs.* < 72.6 g/d Dietary assessment methods: baseline questionnaire with 55-food items, 126-item FFQ, center-specific dietary questionnaires (EPIC), FFQ (block)	Ovarian cancer				Low
	4 cohort studies	- *n* = 464,643 participants - *n* = 1,585 cases	Total protein			0.96 (0.88, 1.06)^2^	*I*^2^ = 0% *P* = 0.741	Low: 5.0	
Mao 2018 [19]	- Published before 09/2017 - Follow-up: 3.5–18 yr	- Only males - Aged 40–92 yr	High *vs.* low protein intake	Total protein: 90–121 g/d *vs.* 0–82 g/d Animal protein: 41–80 g/d *vs.* ≤ 47 g/d Plant protein: 35–47 g/d *vs.* ≤ 29 g/d Dietary assessment methods NP	Prostate cancer				Low
	8 cohort studies	*n* = 265,067 participants *n* = 5,860 cases	Total protein			1.080 (0.964, 1.209)^1^	*I*^2^ = 0% *P* = 0.670	Very low: 2.9	
	4 cohort studies and 1 case–control study	*n* = 220,916 participants *n* = 8,826 cases	Animal protein			1.001 (0.917, 1.092)^1^	*I*^2^ = 0% *P* = 0.891	Very low: 3.5	
	4 cohort studies and 1 case–control study	*n* = 234,462 participants *n* = 8,937 cases	Plant protein			0.986 (0.904, 1.076)^1^	*I*^2^ = 0% *P* = 0.556	Very low: 3.5	
Pang 2018 [20]	- Published until 06/2018 - Follow-up: NP	- Only females - Aged 18–87 yr	High *vs.* low protein intake	> 89.9 g/d *vs.* < 72.6 g/d Dietary assessment methods NP	Ovarian cancer				Low
	2 cohort studies	*n* = 63,275 participants *n* = 210 cases	Total protein			0.903 (0.679, 1.201)^1^	*I*^2^ = 27.9% *P* = 0.239	Very low: 2.5	
Lai 2017 [21]	- Published until 12/2016 - Follow-up: NP	- Both sexes - Aged 20–89 yr	High *vs.* low protein intake	115.9 g/d *vs.* 83.8 g/day dietary assessment methods: Semiquantitative FFQ, FFQ,	Colorectal cancer				High
	3 cohort studies	*n* = 680 cases number of participants: NP	Total protein			0.939 (0.730, 1.209)^1^	*I*^2^ = 0% *P* = 0.980	Low: 4.0	

B) Systematic reviews without meta-analysis								
Author, year	Study type, study period	Study population	Exposition	Protein intake	Outcome	Results	NutriGrade rating	AMSTAR 2 rating
Ubago-Guisado 2021 [17]	- Published until 05/2021 - Follow-up: 8.7 yr	- Only males - Age NP	High *vs.* low protein intake	Protein intake NP Dietary assessment methods NP	Prostate cancer			Moderate
	1 cohort study	*n* = 142,520 participants *n* = 2727 cases	Total protein			*“Total protein intake was not positively associated with increased risk (HR 1.17 (0.96, 1.44)”*	NA	
	1 cohort study	*n* = 142,520 participants *n* = 2727 cases	Dairy protein			*“Protein from dairy foods was significantly associated with an increased risk (HR 1.22 (1.07, 1.41)”* *“An increment of 35 g/d dairy protein was associated with an HR of 1.32 (1.01, 1.72)”*	NA	
Pedersen 2013 [22]	- Published between 01/2000 and 12/2011 - Follow-up: 5.5–18 yr	- Only females - Healthy - Aged 30–70 yr	High *vs.* low protein intake	Protein intake np Dietary assessment methods: 61-item ffq over the past year, 107-item ffq over the previous year	Breast cancer			Moderate
	1 cohort study and 1 nested case–control study	- *n* = 88,647 participants - *n* = 214 controls - *n* = 4,163 cases	Total protein			None of the two studies found statistically significant associations Between total, animal or plant protein and breast cancer risk	Low: 4.0	
	1 cohort study and 1 nested case–control study	- *n* = 88,647 participants - *n* = 214 controls - *n* = 4,163 cases	Animal protein				Low: 4.0	
	1 cohort study and 1 nested case–control study	- *n* = 88,647 participants - *n* = 214 controls - *n* = 4,163 cases	Plant protein				Low: 4.0	

*AMSTAR 2* A Measurement Tool to Assess Systematic Reviews, *CI* confidence interval, *d* day(s) *FFQ* Food-frequency questionnaire, *NA* not applicable, *NP* not provided, *RR* relative risk, *yr* year(s), *HR* hazard ratio

^1^random effect model

^2^fixed effect model

The included SRs comprised 32 primary studies and investigated the following outcomes: prostate cancer [14, 17, 19], ovarian cancer [18, 20], colorectal cancer [21], breast cancer [13, 22], pancreatic cancer [16] and overall cancer incidence [15]. Eight SRs included assessments of total protein in relation to cancer risks [14, 16–22], and separate assessments of plant protein and animal protein were reported in four SRs [14, 16, 19, 22]. Two SRs addressed soy protein and cancer risk [13, 15]. Two SRs investigated dairy protein in relation to cancer risk [14, 17]. The study duration of the included primary studies ranged from 3.5 to 18 years for cohort studies. The SRs on colorectal, pancreatic and overall cancer incidence included both sexes, while the SRs on prostate cancer included only male participants and the SRs on breast and ovarian cancer only included female participants. The age of the participants ranged from 18 to 92 years, and participants were free of cancer at baseline.

### Type and range of protein intake

The type and amount of protein intake was not provided in five SRs [14–17, 22]. For the other five SRs the reported total protein intake ranged between 70 and 129 g/d vs. 0–84 g/d, the range of animal protein intake was 41–80 g/d vs. 9–47 g/d, and the range of plant protein intake was 28–47 g/d vs. 13–29 g/d for cohort studies, although exact minimum and maximum values for protein intakes were not available from all original studies included. Of note, food frequency questionnaires (FFQ) were used to estimate protein intakes in the vast majority of the original studies included in the SRs.

### Methodological quality

Overall scores of AMSTAR 2 for each included SR are reported in Table 2. Supplementary Materials (S7) provide a more detailed overview showing the assessments of each individual item. Methodological quality was rated high for one SR [21], moderate for five SRs [13–15, 17, 22] and low for four SRs [16, 18–20].

### Outcome-specific certainty of the evidence

None of the included SRs found a statistically significant (p < 0.05) association between protein intake and cancer risk and risk estimates comparing highest versus lowest intake categories were largely close to 1. One exception is a statistically significant positive association that was observed between dairy protein intake and prostate cancer risk [14, 17].

Overall scores of NutriGrade for each SR are summarised in Table 2. Briefly, out of the 17 NutriGrade ratings, seven were very low, nine were low, and one was moderate. Supplementary Materials (S8) provides a more detailed account showing the assessments of each individual NutriGrade item.

### Rating of the overall certainty of the evidence

According to our pre-specified criteria [23], the overall evidence for the lack of an association between total protein intake and colorectal cancer and between total, animal and plant protein intake and breast cancer was rated as *possible*. By contrast, the evidence regarding animal and plant protein consumption in relation to colorectal cancer risk was rated as *insufficient*, in view of a *low* methodological quality of the underlying studies. For the same reason, the overall evidence on associations between protein intake (total, animal-derived, and plant-derived) and ovarian and prostate cancer was rated as *insufficient*.

## Discussion

In the present umbrella review, 10 SRs, of which eight also included meta-analyses, were identified to derive the available evidence on the association between dietary protein intake and cancer risks, considering colorectal cancer, breast cancer, ovarian cancer, prostate cancer, pancreatic cancer and overall cancer incidence. Overall, no association between dietary protein intake and risk of cancer was observed. However, the evidence for this lack of an association was only rated as *possible* with regard to total protein intake and colorectal and breast cancer risk, while the evidence for associations between protein intake and the evidence for other cancer types was rated as *insufficient*.

Previous umbrella reviews have been conducted in the field of diet and cancer risk [30–34], but not all of them explicitly included protein intake. An umbrella review on the role of diet on colorectal cancer incidence included dietary protein intake as exposure and referred to the SR by Lai et al. 2017 (which is also included in our umbrella review) considering the association between dietary protein intake as association with non-significant evidence with respect to colorectal cancer incidence [30].

The studies included in the here reviewed SRs and meta-analyses assessed dietary intake mainly by FFQs, which are prone to misclassification bias and limited in determining absolute intake values, but have been shown to be appropriate to rank study participants according to their dietary intakes [35, 36]. The use of different nutrient databases for the calculation of protein intake from food intake may have introduced additional heterogeneity and misclassification. However, we included here SRs that are largely based on cohort studies, in which the misclassification due to the dietary assessment is often non-differential since the exposure is measured before the outcome [37]. Nevertheless, such non-differential misclassification is particularly expected for protein intake estimated from FFQs as compared to estimation of fat and carbohydrate intake [38] and may have led to severe attenuation of estimated protein intake-cancer relationships impairing the detection of small associations [39]. In addition, analyses on animal versus plant protein intake may be especially prone to residual confounding due to associations with other lifestyle factors that may be related to cancer risk. Finally, it cannot be excluded that other co-occurring ingredients in protein-rich foods may play a role in the observed associations.

It has been suggested that high protein intake may be related to cancer risk through alterations in the IGF-1 axis. IGF-1 physiologically regulates cellular proliferation, differentiation, and apoptosis [40], but may also exert mitogenic and anti-apoptotic effects that promote carcinogenesis [41, 42]. High circulating IGF-1 concentrations have been associated with cancer risk [43], recently supported by Mendelian randomisation studies, particularly for breast, colorectal and prostate cancer [44–47], while no significant associations were observed for ovarian cancer [45], and no corresponding evidence is available for pancreatic cancer. There is evidence that high protein intake is associated with higher circulating IGF-1 from intervention studies [48], as well as from observational studies [49–57], although some smaller observational studies did not observe significant associations [58–62]. There is also evidence that higher animal [49], and particularly dairy protein intake [49, 55, 56] is associated with higher circulating IGF-1 [60]. However, it is questionable whether the impact of protein intake on IGF-1 concentrations is sufficient to observe altered cancer risks on a population level. For example, in a recent investigation within the UK Biobank comparing the highest (mean 89.8 g/day) versus lowest (mean 68.1 g/day) quintile of total protein intake was associated with 1.72 nmol/L higher IGF-1 concentrations [57]. However, based on the Mendelian Randomisation analysis on cancer in the UK Biobank [45], such a difference would translate to only 1% differences in risk of colorectal (OR per 5.7 nmol/L higher IGF-1 was 1.11, 95% CI 1.01–1.22) or prostate cancer (OR per 5.7 nmol/L higher IGF-1 was RR 1.10; 95% CI 1.01–1.21).

Besides prospective studies investigating protein intake on the macronutrient level in relation to cancer risk, numerous studies have been conducted on the association between protein-rich foods and cancer risk. For example, dairy and especially milk intake has been related to moderately higher risk of prostate cancer, and it has been hypothesised that the underlying mechanism is through dairy protein intake increasing circulating IGF-1 [63]. However, a recent Mendelian randomisation study found only limited evidence for an association between genetically determined higher milk intake (proxied through a genetic variant associated with lactase persistence in adulthood) and risk of prostate cancer [64]. In the two SRs with meta-analysis on prostate cancer included in the present umbrella review [14, 19], no associations were observed for total protein, plant or animal protein intake. In the more recent SR with meta-analysis on prostate cancer, a significant positive association between dairy protein intake and prostate cancer was observed. In contrast to the observed positive association with prostate cancer, dairy intake has been consistently associated with lower risk of colorectal cancer, recently confirmed by a Mendelian randomisation study [64], but the hypothesised mechanism is here not through dairy protein, but through dairy calcium [65, 66]. Another protein source that has been consistently associated with risk of colorectal cancer is meat intake [67]. However, since positive associations were observed with red and processed meat intake and no or inverse associations with white meat intake [68], the underlying mechanism may be unrelated to protein. In line with this suggestion, red and processed meat intake have been shown to be only weakly associated with IGF-1 concentration [69]. Heme iron and compounds formed during the preparation such as heterocyclic amines, polycyclic aromatic hydrocarbons, as well as nitrate and N-nitroso-compounds, have been suggested here as underlying mechanisms for the observed positive associations between red and processed meat and prostate cancer risk [70, 71]. The SR with meta-analysis [21] included in this umbrella review did not observe an association between total protein intake and risk of colorectal cancer summarising data from three cohort studies, and no association for animal (5 case–control or cohort studies) or plant (4 case–control or cohort studies) protein intake. Dairy intake has also been related to lower risk of breast cancer [72, 73], but a recent pooled analysis of cohort studies comprising more than one million women did not find associations [74], and also the evidence from Mendelian randomisation studies was inconclusive [64].

Protein sources from plant origin such as legumes [75] have been inconclusively related to cancer risk, while soy intake has been related to lower risk of breast cancer [76], although the suggested underlying mechanism is ascribed to phytoestrogens, and thus probably unrelated to protein per se. The SRs with meta-analysis on soy protein intake included here did not observe an association with overall cancer incidence [15] or breast cancer risk [13].

We acknowledge that through alterations in the IGF-1 system there is a plausible biological mechanism that may mediate positive associations between protein intake and cancer risk although the quantitative importance may be limited. At the same time, other nutrients that co-occur with protein in food sources, such as calcium in milk, which may reduce colorectal cancer risk, and heme iron and heterocyclic amines in red meat and N-nitroso-compounds in processed meat, which may increase colorectal cancer risk, as well as phytoestrogens in soy, which may reduce breast cancer risk, may mask potential associations with overall protein intake. This may explain why on the macronutrient level protein intake has not been consistently related to cancer risk.

Strengths of the current umbrella review include the comprehensive literature search, as well as the assessment of the methodological quality of the included SRs with AMSTAR 2 and the rating of their outcome-specific certainty of evidence with NutriGrade. We applied NutriGrade instead of the GRADE approach (Grading of Recommendations, Assessment, Development and Evaluation) because an important novelty of NutriGrade (published in 2016) was the modified classification for meta-analyses of RCTs and cohort studies compared with the traditional GRADE approach (initially classifying RCTs with an initial high score and cohort studies with a low score) [77]. We are aware that in the meantime the GRADE approach was amended (adjustments published in 2019, but after the guideline methodology was established in 2017) in a way that cohort studies can now also be assigned an initially high score, when risk of bias tools such as ROBINS-I are used [78]. A general limitation of this umbrella review is that all included SRs were based on observational studies where dietary assessment was usually performed through FFQs, thereby limiting the ability to draw conclusions regarding absolute intakes and to detect small associations due to measurement error. Only a limited number of SRs were available for inclusion in the present umbrella review, and for certain types of cancer (e.g. lung cancer, kidney cancer, bladder cancer), no SRs were available. The scarcity of SRs on the topic may also be related to the fact that protein intake is more rarely investigated in prospective cohort studies than food intake. A further limitation is the limited generalizability of results given that the prospective cohort studies included in the here reviewed SRs are usually not representative of the general population. In addition, we cannot exclude that the associations observed in the included SRs were subject to residual confounding due to insufficient confounder adjustment in the original studies.

## Conclusion

The present umbrella review concludes that there is no association between total protein intake and risk of colorectal cancer and between total, animal and plant protein intake and risk of breast cancer, at *possible* certainty of the evidence. The certainty of the evidence is *insufficient* with regard to intakes of animal- and plant-derived protein in relation to colorectal cancer. Moreover, the certainty of evidence for the lack of associations between protein intake (total, animal-derived and plant-derived) and risks of breast, ovarian, and prostate cancer, as well as overall cancer incidence from the identified SRs, is also *insufficient*. Thus, unless stronger evidence from prospective studies becomes available, our overall finding that protein intake may not be associated with cancer risk needs to be interpreted with caution.

### Supplementary Information

Below is the link to the electronic supplementary material.

Supplementary file1 (DOCX 32 KB)

Supplementary file2 (DOCX 16 KB)

Supplementary file3 (DOCX 48 KB)

Supplementary file4 (DOCX 46 KB)

Supplementary file5 (XLSX 11 KB)

Supplementary file6 (DOCX 28 KB)

Supplementary file7 (DOCX 21 KB)

Supplementary file8 (XLSX 26 KB)

Supplementary file9 (XLSX 14 KB)

## Data Availability

Not applicable.

## Abbreviations

AMSTAR 2: A Measurement Tool to Assess Systematic Reviews 2
FFQs: Food frequency questionnaires
IGF-1: Insulin-like growth factor 1
RCT: Randomised controlled trial
SR: Systematic review
WCRF: World Cancer Research Fund

## References

[1] Bray F, Ferlay J, Soerjomataram I et al (2018) Global cancer statistics 2018: GLOBOCAN estimates of incidence and mortality worldwide for 36 cancers in 185 countries. CA Cancer J Clin 68:394–424. 10.3322/caac.21492 30207593 10.3322/caac.21492

[2] WHO (World Health Organization) Cancer. Key Facts. https://www.who.int/news-room/fact-sheets/detail/cancer. Accessed 31 Dec 2020

[3] American Cancer Society (2020) The Cancer Atlas. Survivorship. 10.3945/ajcn.115.112086 10.3945/ajcn.115.112086

[4] GBD (2019) Cancer Risk Factors Collaborators (2022) The global burden of cancer attributable to risk factors, 2010–19: a systematic analysis for the Global Burden of Disease Study 2019. Lancet 400:563–591. 10.1016/S0140-6736(22)01438-6 10.1016/S0140-6736(22)01438-6 PMC9395583 35988567

[5] Brown KF, Rumgay H, Dunlop C et al (2018) The fraction of cancer attributable to modifiable risk factors in England, Wales, Scotland, Northern Ireland, and the United Kingdom in 2015. Br J Cancer 118:1130–1141. 10.1038/s41416-018-0029-6 29567982 10.1038/s41416-018-0029-6 PMC5931106

[6] Islami F, Goding Sauer A, Miller KD et al (2018) Proportion and number of cancer cases and deaths attributable to potentially modifiable risk factors in the United States. CA Cancer J Clin 68:31–54. 10.3322/caac.21440 29160902 10.3322/caac.21440

[7] Mons U, Gredner T, Behrens G et al (2018) Cancers due to smoking and high alcohol consumption. Dtsch Arztebl Int 115:571–577. 10.3238/arztebl.2018.0571 30236215 10.3238/arztebl.2018.0571 PMC6206255

[8] Behrens G, Gredner T, Stock C et al (2018) Cancers due to excess weight, low physical activity, and unhealthy diet. Dtsch Arztebl Int 115:578–585. 10.3238/arztebl.2018.0578 30236216 10.3238/arztebl.2018.0578 PMC6206246

[9] Cascella M, Bimonte S, Barbieri A et al (2018) Dissecting the mechanisms and molecules underlying the potential carcinogenicity of red and processed meat in colorectal cancer (CRC): an overview on the current state of knowledge. Infect Agent Cancer 13:3. 10.1186/s13027-018-0174-9 29371880 10.1186/s13027-018-0174-9 PMC5769331

[10] Levine ME, Suarez JA, Brandhorst S et al (2014) Low Protein Intake Is Associated with a Major Reduction in IGF-1, Cancer, and Overall Mortality in the 65 and Younger but Not Older Population. Cell Metab 19:407–417. 10.1016/j.cmet.2014.02.006 24606898 10.1016/j.cmet.2014.02.006 PMC3988204

[11] Klement RJ, Fink MK (2016) Dietary and pharmacological modification of the insulin/IGF-1 system: exploiting the full repertoire against cancer. Oncogenesis 5:e193. 10.1038/oncsis.2016.2 26878387 10.1038/oncsis.2016.2 PMC5154349

[12] Yin J, Ren W, Huang X et al (2018) Protein restriction and cancer. Biochim Biophys Acta Rev Cancer 1869:256–262. 10.1016/j.bbcan.2018.03.004 29596961 10.1016/j.bbcan.2018.03.004

[13] Shin S, Fu J, Shin W-K et al (2023) Association of food groups and dietary pattern with breast cancer risk: a systematic review and meta-analysis. Clin Nutr 42:282–297. 10.1016/j.clnu.2023.01.003 36731160 10.1016/j.clnu.2023.01.003

[14] Alzahrani MA, Shakil Ahmad M, Alkhamees M et al (2022) Dietary protein intake and prostate cancer risk in adults: a systematic review and dose-response meta-analysis of prospective cohort studies. Complement Ther Med. 10.1016/j.ctim.2022.102851 35820576 10.1016/j.ctim.2022.102851

[15] Fan Y, Wang M, Li Z et al (2022) Intake of soy, soy isoflavones and soy protein and risk of cancer incidence and mortality. Front Nutr. 10.3389/fnut.2022.847421 35308286 10.3389/fnut.2022.847421 PMC8931954

[16] Zhang T, Wu S, Xu F et al (2022) The association between dietary protein intake and the risk of pancreatic cancer: evidence from 14 publications. Nutr Cancer 74:3172–3178. 10.1080/01635581.2022.2059529 35414283 10.1080/01635581.2022.2059529

[17] Ubago-Guisado E, Rodríguez-Barranco M, Ching-López A et al (2021) Evidence update on the relationship between diet and the most common cancers from the European Prospective Investigation into Cancer and Nutrition (EPIC) Study: a systematic review. Nutrients 13:3582. 10.3390/nu13103582 34684583 10.3390/nu13103582 PMC8540388

[18] Khodavandi A, Alizadeh F, Razis AFA (2021) Association between dietary intake and risk of ovarian cancer: a systematic review and meta-analysis. Eur J Nutr 60:1707–1736. 10.1007/s00394-020-02332-y 32661683 10.1007/s00394-020-02332-y

[19] Mao Y, Tie Y, Du J (2018) Association between dietary protein intake and prostate cancer risk: evidence from a meta-analysis. World J Surg Oncol 16:152. 10.1186/s12957-018-1452-0 30041648 10.1186/s12957-018-1452-0 PMC6058353

[20] Pang Y, Wang W (2018) Dietary protein intake and risk of ovarian cancer: evidence from a meta-analysis of observational studies. Biosci Rep. 10.1042/BSR20181857 10.1042/BSR20181857 PMC6294619 30401730

[21] Lai R, Bian Z, Lin H et al (2017) The association between dietary protein intake and colorectal cancer risk: a meta-analysis. World J Surg Oncol 15:169. 10.1186/s12957-017-1241-1 28886717 10.1186/s12957-017-1241-1 PMC5591555

[22] Pedersen AN, Kondrup J, Børsheim E (2013) Health effects of protein intake in healthy adults: a systematic literature review. Food Nutr Res 57:21245. 10.3402/fnr.v57i0.21245 10.3402/fnr.v57i0.21245 PMC3730112 23908602

[23] Kroke A, Schmidt A, Amini AM et al (2022) Dietary protein intake and health-related outcomes: a methodological protocol for the evidence evaluation and the outline of an evidence to decision framework underlying the evidence-based guideline of the German Nutrition Society. Eur J Nutr 61:2091–2101. 10.1007/s00394-021-02789-5 35031889 10.1007/s00394-021-02789-5 PMC9106629

[24] Shea BJ, Reeves BC, Wells G et al (2017) AMSTAR 2: a critical appraisal tool for systematic reviews that include randomised or non-randomised studies of healthcare interventions, or both. BMJ. 10.1136/bmj.j4008 28935701 10.1136/bmj.j4008 PMC5833365

[25] Schwingshackl L, Knüppel S, Schwedhelm C et al (2016) Perspective: NutriGrade: A scoring system to assess and judge the meta-evidence of randomized controlled trials and cohort studies in nutrition research. Adv Nutr 7:994–1004. 10.3945/an.116.013052 28140319 10.3945/an.116.013052 PMC5105044

[26] Alonso-Coello P, Schünemann HJ, Moberg J et al. (2016) GRADE Evidence to Decision (EtD) frameworks: a systematic and transparent approach to making well informed healthcare choices. 1: Introduction. BMJ 353:i2016. doi: 10.1136/bmj.i2016 10.1136/bmj.i2016 27353417

[27] Gathirua-Mwangi WG, Zhang J (2014) Dietary factors and risk for advanced prostate cancer. Eur J Cancer Prev 23:96–109. 10.1097/CEJ.0b013e3283647394 23872953 10.1097/CEJ.0b013e3283647394 PMC4091618

[28] WCRF (World Cancer Research Fund), AICR (American Institute for Cancer Research) (2015) The associations between diet, nutrition and physical activity and the risk of kidney cancer. Continuous update project. https://www.wcrf.org/wp-content/uploads/2021/02/kidney-cancer-slr.pdf. Accessed 07 Dec 2022

[29] Wu SH, Liu Z (2013) Soy food consumption and lung cancer risk: a meta-analysis using a common measure across studies. Nutr Cancer 65:625–632. 10.1080/01635581.2013.795983 23859029 10.1080/01635581.2013.795983 PMC3858084

[30] Veettil SK, Wong TY, Loo YS et al (2021) Role of diet in colorectal cancer incidence: umbrella review of meta-analyses of prospective observational studies. JAMA Netw Open. 10.1001/jamanetworkopen.2020.37341 33591366 10.1001/jamanetworkopen.2020.37341 PMC7887658

[31] Sun H, Gong T-T, Xia Y et al (2021) Diet and ovarian cancer risk: an umbrella review of systematic reviews and meta-analyses of cohort studies. Clin Nutr 40:1682–1690. 10.1016/j.clnu.2020.11.032 33308841 10.1016/j.clnu.2020.11.032

[32] Papadimitriou N, Markozannes G, Kanellopoulou A et al (2021) An umbrella review of the evidence associating diet and cancer risk at 11 anatomical sites. Nat Commun. 10.1038/s41467-021-24861-8 34321471 10.1038/s41467-021-24861-8 PMC8319326

[33] Buja A, Pierbon M, Lago L et al (2020) Breast cancer primary prevention and diet: an umbrella review. Int J Environ Res Public Health 17:4731. 10.3390/ijerph17134731 32630215 10.3390/ijerph17134731 PMC7369836

[34] Markozannes G, Tzoulaki I, Karli D et al (2016) Diet, body size, physical activity and risk of prostate cancer: an umbrella review of the evidence. Eur J Cancer 69:61–69. 10.1016/j.ejca.2016.09.026 27816833 10.1016/j.ejca.2016.09.026

[35] Willett WC, Sampson L, Stampfer MJ et al (1985) Reproducibility and validity of a semiquantitative food frequency questionnaire. Am J Epidemiol 122:51–65. 10.1093/oxfordjournals.aje.a114086 4014201 10.1093/oxfordjournals.aje.a114086

[36] Subar AF, Kushi LH, Lerman JL et al (2017) Invited commentary: the contribution to the field of nutritional epidemiology of the landmark 1985 publication by Willett et al. Am J Epidemiol 185:1124–1129 28535308 10.1093/aje/kwx072

[37] Kipnis V, Freedman LS (2008) Impact of exposure measurement error in nutritional epidemiology. J Natl Cancer Inst 100:1658–1659. 10.1093/jnci/djn408 19033567 10.1093/jnci/djn408

[38] Yuan C, Spiegelman D, Rimm EB et al (2017) Validity of a dietary questionnaire assessed by comparison with multiple weighed dietary records or 24-hour recalls. Am J Epidemiol 185:570–584. 10.1093/aje/kww104 28338828 10.1093/aje/kww104 PMC5859994

[39] Kipnis V, Subar AF, Midthune D et al (2003) Structure of dietary measurement error: results of the OPEN biomarker study. Am J Epidemiol 158:14–21. 10.1093/aje/kwg091 12835281 10.1093/aje/kwg091

[40] Jones JI, Clemmons DR (1995) Insulin-like growth factors and their binding proteins: biological actions. Endocr Rev 16:3–34. 10.1210/edrv-16-1-3 7758431 10.1210/edrv-16-1-3

[41] Pollak M (2012) The insulin and insulin-like growth factor receptor family in neoplasia: an update. Nat Rev Cancer 12:159–169. 10.1038/nrc3215 22337149 10.1038/nrc3215

[42] Valentinis B, Baserga R (2001) IGF-I receptor signalling in transformation and differentiation. Mol Pathol 54:133–137. 10.1136/mp.54.3.133 11376123 10.1136/mp.54.3.133 PMC1187050

[43] Nimptsch K, Pischon T (2016) Obesity biomarkers, metabolism and risk of cancer: an epidemiological perspective. Recent Results Cancer Res 208:199–217. 10.1007/978-3-319-42542-9_11 27909909 10.1007/978-3-319-42542-9_11

[44] Watts EL, Fensom GK, Smith Byrne K et al (2021) Circulating insulin-like growth factor-I, total and free testosterone concentrations and prostate cancer risk in 200 000 men in UK Biobank. Int J Cancer 148:2274–2288. 10.1002/ijc.33416 33252839 10.1002/ijc.33416 PMC8048461

[45] Larsson SC, Carter P, Vithayathil M et al (2020) Insulin-like growth factor-1 and site-specific cancers: a Mendelian randomization study. Cancer Med 9:6836–6842. 10.1002/cam4.3345 32717139 10.1002/cam4.3345 PMC7520358

[46] Murphy N, Knuppel A, Papadimitriou N et al (2020) Insulin-like growth factor-1, insulin-like growth factor-binding protein-3, and breast cancer risk: observational and Mendelian randomization analyses with ∼430 000 women. Ann Oncol 31:641–649. 10.1016/j.annonc.2020.01.066 32169310 10.1016/j.annonc.2020.01.066 PMC7221341

[47] Murphy N, Carreras-Torres R, Song M et al (2020) Circulating levels of insulin-like growth factor 1 and insulin-like growth factor binding protein 3 associate with risk of colorectal cancer based on serologic and Mendelian randomization analyses. Gastroenterology 158:1300-1312.e20. 10.1053/j.gastro.2019.12.020 31884074 10.1053/j.gastro.2019.12.020 PMC7152801

[48] Kazemi A, Speakman JR, Soltani S et al (2020) Effect of calorie restriction or protein intake on circulating levels of insulin like growth factor I in humans: a systematic review and meta-analysis. Clin Nutr 39:1705–1716. 10.1016/j.clnu.2019.07.030 31431306 10.1016/j.clnu.2019.07.030

[49] Crowe FL, Key TJ, Allen NE et al (2009) The association between diet and serum concentrations of IGF-I, IGFBP-1, IGFBP-2, and IGFBP-3 in the European Prospective Investigation into Cancer and Nutrition. Cancer Epidemiol Biomarkers Prev 18:1333–1340. 10.1158/1055-9965.EPI-08-0781 19423514 10.1158/1055-9965.EPI-08-0781

[50] Giovannucci E, Pollak M, Liu Y et al (2003) Nutritional predictors of insulin-like growth factor I and their relationships to cancer in men. Cancer Epidemiol Biomarkers Prev 12:84–89 12582016

[51] Holmes MD, Pollak MN, Willett WC et al (2002) Dietary correlates of plasma insulin-like growth factor I and insulin-like growth factor binding protein 3 concentrations. Cancer Epidemiol Biomarkers Prev 11:852–861 12223429

[52] Larsson SC, Wolk K, Brismar K et al (2005) Association of diet with serum insulin-like growth factor I in middle-aged and elderly men. Am J Clin Nutr 81:1163–1167. 10.1093/ajcn/81.5.1163 15883443 10.1093/ajcn/81.5.1163

[53] McGreevy KM, Hoel BD, Lipsitz SR et al (2007) Impact of nutrients on insulin-like growth factor-I, insulin-like growth factor binding protein-3 and their ratio in African American and white males. Public Health Nutr 10:97–105. 10.1017/S1368980007217999 17212848 10.1017/S1368980007217999

[54] Norat T, Dossus L, Rinaldi S et al (2007) Diet, serum insulin-like growth factor-I and IGF-binding protein-3 in European women. Eur J Clin Nutr 61:91–98. 10.1038/sj.ejcn.1602494 16900085 10.1038/sj.ejcn.1602494

[55] Young NJ, Metcalfe C, Gunnell D et al (2012) A cross-sectional analysis of the association between diet and insulin-like growth factor (IGF)-I, IGF-II, IGF-binding protein (IGFBP)-2, and IGFBP-3 in men in the United Kingdom. Cancer Causes Control 23:907–917. 10.1007/s10552-012-9961-6 22527168 10.1007/s10552-012-9961-6

[56] Bradbury KE, Balkwill A, Tipper SJ et al (2015) The association of plasma IGF-I with dietary, lifestyle, anthropometric, and early life factors in postmenopausal women. Growth Horm IGF Res Off J Growth Horm Res Soc Internat IGF Res Soc 25:90–95. 10.1016/j.ghir.2015.01.001 10.1016/j.ghir.2015.01.001 25641638

[57] Watling CZ, Kelly RK, Tong TYN et al (2021) Associations of circulating insulin-like growth factor-I with intake of dietary proteins and other macronutrients. Clin Nutr 40:4685–4693. 10.1016/j.clnu.2021.04.021 34237695 10.1016/j.clnu.2021.04.021 PMC8345002

[58] DeLellis K, Rinaldi S, Kaaks RJ et al (2004) Dietary and lifestyle correlates of plasma insulin-like growth factor-I (IGF-I) and IGF binding protein-3 (IGFBP-3): the multiethnic cohort. Cancer Epidemiol Biomarkers Prev 13:1444–1451 15342444 10.1158/1055-9965.1444.13.9

[59] Gunnell D, Oliver SE, Peters TJ et al (2003) Are diet-prostate cancer associations mediated by the IGF axis? A cross-sectional analysis of diet, IGF-I and IGFBP-3 in healthy middle-aged men. Br J Cancer 88:1682–1686. 10.1038/sj.bjc.6600946 12771980 10.1038/sj.bjc.6600946 PMC2377147

[60] Beasley JM, Gunter MJ, LaCroix AZ et al (2014) Associations of serum insulin-like growth factor-I and insulin-like growth factor-binding protein 3 levels with biomarker-calibrated protein, dairy product and milk intake in the Women‘s Health Initiative. Br J Nutr 111:847–853. 10.1017/S000711451300319X 24094144 10.1017/S000711451300319X PMC3978780

[61] Mucci LA, Tamimi R, Lagiou P et al (2001) Are dietary influences on the risk of prostate cancer mediated through the insulin-like growth factor system? BJU Int 87:814–820. 10.1046/j.1464-410x.2001.02191.x 11412218 10.1046/j.1464-410x.2001.02191.x

[62] Signorello LB, Kuper H, Lagiou P et al (2000) Lifestyle factors and insulin-like growth factor 1 levels among elderly men. Eur J Cancer Prev 9:173–178 10954256 10.1097/00008469-200006000-00004

[63] Harrison S, Lennon R, Holly J et al (2017) Does milk intake promote prostate cancer initiation or progression via effects on insulin-like growth factors (IGFs)? A systematic review and meta-analysis. Cancer Causes Control 28:497–528. 10.1007/s10552-017-0883-1 28361446 10.1007/s10552-017-0883-1 PMC5400803

[64] Larsson SC, Mason AM, Kar S et al (2020) Genetically proxied milk consumption and risk of colorectal, bladder, breast, and prostate cancer: a two-sample Mendelian randomization study. BMC Med 18:370. 10.1186/s12916-020-01839-9 33261611 10.1186/s12916-020-01839-9 PMC7709312

[65] Newmark HL, Wargovich MJ, Bruce WR (1984) Colon cancer and dietary fat, phosphate, and calcium: a hypothesis. J Natl Cancer Inst 72:1323–1325 6587152

[66] Lamprecht SA, Lipkin M (2001) Cellular mechanisms of calcium and vitamin D in the inhibition of colorectal carcinogenesis. Ann N Y Acad Sci 952:73–87. 10.1111/j.1749-6632.2001.tb02729.x 11795445 10.1111/j.1749-6632.2001.tb02729.x

[67] Zhao Z, Feng Q, Yin Z et al (2017) Red and processed meat consumption and colorectal cancer risk: a systematic review and meta-analysis. Oncotarget 8:83306–83314 29137344 10.18632/oncotarget.20667 PMC5669970

[68] Lippi G, Mattiuzzi C, Cervellin G (2016) Meat consumption and cancer risk: a critical review of published meta-analyses. Crit Rev Oncol Hematol 97:1–14. 10.1016/j.critrevonc.2015.11.008 26633248 10.1016/j.critrevonc.2015.11.008

[69] Watling CZ, Kelly RK, Tong TYN et al (2023) Associations between food group intakes and circulating insulin-like growth factor-I in the UK Biobank: a cross-sectional analysis. Eur J Nutr 62:115–124. 10.1007/s00394-022-02954-4 35906357 10.1007/s00394-022-02954-4 PMC9899744

[70] Bastide NM, Pierre FHF, Corpet DE (2011) Heme iron from meat and risk of colorectal cancer: A meta-analysis and a review of the mechanisms involved. Cancer Prev Res (Phila) 4:177–184. 10.1158/1940-6207.CAPR-10-0113 21209396 10.1158/1940-6207.CAPR-10-0113

[71] Bouvard V, Loomis D, Guyton KZ et al (2015) Carcinogenicity of consumption of red and processed meat. Lancet Oncol 16:1599–1600. 10.1016/S1470-2045(15)00444-1 26514947 10.1016/S1470-2045(15)00444-1

[72] He Y, Tao Q, Zhou F et al (2021) The relationship between dairy products intake and breast cancer incidence: a meta-analysis of observational studies. BMC Cancer 21:1109. 10.1186/s12885-021-08854-w 34654387 10.1186/s12885-021-08854-w PMC8520314

[73] Dong JY, Zhang L, He K et al (2011) Dairy consumption and risk of breast cancer: a meta-analysis of prospective cohort studies. Breast Cancer Res Treat 127:23–31. 10.1007/s10549-011-1467-5 21442197 10.1007/s10549-011-1467-5

[74] Wu Y, Huang R, Wang M et al (2021) Dairy foods, calcium, and risk of breast cancer overall and for subtypes defined by estrogen receptor status: a pooled analysis of 21 cohort studies. Am J Clin Nutr 114:450–461. 10.1093/ajcn/nqab097 33964859 10.1093/ajcn/nqab097 PMC8326053

[75] Martini D, Godos J, Marventano S et al (2021) Nut and legume consumption and human health: an umbrella review of observational studies. Int J Food Sci Nutr 72:871–878. 10.1080/09637486.2021.1880554 33541169 10.1080/09637486.2021.1880554

[76] Kazemi A, Barati-Boldaji R, Soltani S et al (2021) Intake of various food groups and risk of breast cancer: a systematic review and dose-response meta-analysis of prospective studies. Adv Nutr 12:809–849. 10.1093/advances/nmaa147 33271590 10.1093/advances/nmaa147 PMC8166564

[77] Schwingshackl L, Schünemann HJ, Meerpohl JJ (2020) Improving the trustworthiness of findings from nutrition evidence syntheses: assessing risk of bias and rating the certainty of evidence. Eur J Nutr. 10.1007/s00394-020-02464-1 33377996 10.1007/s00394-020-02464-1 PMC8354882

[78] Schünemann HJ, Cuello C, Akl EA et al (2019) GRADE guidelines: 18. How ROBINS-I and other tools to assess risk of bias in nonrandomized studies should be used to rate the certainty of a body of evidence. J Clin Epidemiol 111:105–114. 10.1016/j.jclinepi.2018.01.012 29432858 10.1016/j.jclinepi.2018.01.012 PMC6692166

