# Supplementary material for: Protein intake and cancer: an umbrella review of systematic reviews for the evidence-based guideline of the German Nutrition Society
Source: Eur J Nutr. 2024 Apr 21;63(5):1471–86. doi: 10.1007/s00394-024-03380-4 (PMC11329548; doi:10.1007/s00394-024-03380-4)
Supplement: Supplementary file 1 — Supplementary file1 (DOCX 32 KB) [file 394_2024_3380_MOESM1_ESM.docx]

**Supplementary Material 3.** Modified AMSTAR 2 questionnaire.

This supplement provides the modified version of the AMSTAR 2 questionnaire that was used to assess the methodological quality of systematic reviews. Please refer to the following publication for the original AMSTAR 2 tool: Shea BJ, Reeves BC, Wells G, Thuku M, Hamel C, Moran J, Moher D, Tugwell P, Welch V, Kristjansson E, Henry DA: AMSTAR 2: a critical appraisal tool for systematic reviews that include randomised or non-randomised studies of healthcare interventions, or both. BMJ 2017;358:j4008.

| **1. Did the research questions and inclusion criteria for the review include the components of PICO?** | | | |
| --- | --- | --- | --- |
| For Yes, ALL the following: | | | |
|  | Population  Intervention  Comparator group  Outcome |  | Yes  No |
| **2. Did the report of the review contain an explicit statement that the review methods were established prior to the conduct of the review and did the report justify any significant deviations from the protocol?** | | | |
| For Yes:  The authors state that they had a written protocol or guide that includes ALL the following: | | | |
|  | review question(s)  a search strategy  inclusion/exclusion criteria  a risk of bias assessment |  | Yes  No |
| **3. Did the review authors use a comprehensive literature search strategy?** | | | |
| For Yes, ALL the following: | | | |
|  | searched at least two databases  (relevant to research question)  provided key word and/or search strategy |  | Yes  No |
| **4. Did the review authors perform study selection in duplicate?** | | | |
| For Yes, either ONE of the following: | | | |
|  | at least two reviewers independently agreed on selection of eligible studies and achieved consensus on which studies to include  OR two reviewers selected a sample of eligible studies and achieved good agreement (at least 80 percent), with the remainder selected by one reviewer |  | Yes  No |

| **5. Did the review authors perform data extraction in duplicate?** | | | | |
| --- | --- | --- | --- | --- |
| For Yes, either ONE of the following: | | | | |
|  | at least two reviewers achieved consensus on which  data to extract from included studies  OR two reviewers selected a sample of eligible studies and achieved good agreement (at least 80 percent), with the remainder selected by one reviewer | |  | Yes  No |
| **6. Did the review authors provide a list of excluded studies and justify the exclusions?** | | | | |
| For Yes: | | | | |
|  | | Provided a flow chart showing the number of excluded studies and reasons for exclusion. A study-specific list is not required. |  | Yes  No |
| **7. Did the review authors describe the included studies in adequate detail?** | | | | |
| For Yes, ALL the following: | | | | |
|  | described population  described interventions  described comparators  described outcomes  described research designs | |  | Yes  No |
| **8. Did the review authors use a satisfactory technique for assessing the risk of bias (RoB) in individual studies that were included in the review?** | | | | |
| For Yes: | | | | |
|  | A tool was used to evaluate the risk of bias of included studies | |  | Yes  No |
| **9. If meta-analysis was performed did the review authors use appropriate methods for statistical combination of results?** | | | | |
| For Yes: | | | | |
|  | Statistical heterogeneity between trial results was assessed and the results are provided | |  | Yes  No  No meta-analysis conducted |

| **10. Did the review authors account for RoB in individual studies when interpreting/discussing the results of the review?** | | | |
| --- | --- | --- | --- |
| For Yes: | | | |
|  | Study quality is considered in the discussion and interpretation |  | Yes  No |
| **11. Did the review authors provide a satisfactory explanation for, and discussion of, any heterogeneity observed in the results of the review?** | | | |
| For Yes, either ONE of the following: | | | |
|  | There was no significant heterogeneity in the results  OR if heterogeneity was present the authors performed an investigation of sources of any heterogeneity in the results and discussed the impact of this on the results of the review |  | Yes  No  No meta-analysis conducted |
| **12. If they performed quantitative synthesis did the review authors carry out an adequate investigation of publication bias (small study bias)?** | | | |
| For Yes: | | | |
|  | Performed graphical or statistical tests for publication bias or an adequate reason is provided for not performing a test. |  | Yes  No  No meta-analysis conducted |
| **13. Did the review authors discuss the likely impact of publication bias on the results of the review?** | | | |
| For Yes, either ONE of the following: | | | |
|  | There was no significant publication bias  OR if publication bias was present the authors discussed the likelihood and magnitude of impact of publication bias on the results of the review |  | Yes  No  No meta-analysis conducted |
| **14. Did the review authors report any potential sources of conflict of interest, including any funding they received for conducting the review?** | | | |
| For Yes, either ONE of the following: | | | |
|  | The authors reported no competing interests  OR The authors described their funding sources and how they managed potential conflicts of interest |  | Yes  No |

Critical assessment items are underlined.

Overall rating

Critical weakness(es):

Non-critical weakness(es):

Rating overall confidence in the results of the review:
