# Supplementary material for: Protein intake and cancer: an umbrella review of systematic reviews for the evidence-based guideline of the German Nutrition Society
Source: Eur J Nutr. 2024 Apr 21;63(5):1471–86. doi: 10.1007/s00394-024-03380-4 (PMC11329548; doi:10.1007/s00394-024-03380-4)
Supplement: Supplementary file 4 — Supplementary file4 (DOCX 46 KB) [file 394_2024_3380_MOESM4_ESM.docx]

**Supplementary Material S5.** Adapted version of the NutriGrade scoring tool.

This supplement provides an overview of the adapted version of the NutriGrade scoring system. The original tool is: Schwingshackl L, Knüppel S, Schwedhelm C, Hoffmann G, Missbach B, Stelmach-Mardas M, Dietrich S, Eichelmann F, Kontopanteils E, Iqbal K, Aleksandrova K, Lorkowski S, Leitzmann MF, Kroke A, Boeing H: Perspective: NutriGrade: A scoring system to assess and judge the meta-evidence of randomized controlled trials and cohort studies in nutrition research. Adv Nutr 2016;7:994–1004.

NutriGrade scoring system for SRs without MA of RCTs

1. Risk of bias/ study quality/ study limitations **(3 P)**
   1. No quantitative and descriptive information available (0 P)
   2. Risk of bias (3 P)
      1. Sequence generation^1^
      2. Allocation concealment^1^
      3. Blinding of participants and personnel^1^
      4. Blinding of outcome assessment personnel^1^
      5. Incomplete outcome^1^
      6. Selective reporting^1^
   3. Study quality (2 P)^2^
2. Precision **(1 P)**
   1. <400 participants (0 P)
   2. ≥400 participants (1 P)
3. Heterogeneity **(1 P)**
   1. >1/3 of included studies have an inconsistent result (i.e. point estimates and/or 95% CI did not overlap between studies) (0 P)
   2. ≥2/3 of included studies have a consistent result (i.e. point estimates and/or 95% CI did overlap between studies) (1 P)
4. Directness **(1 P)**
   1. Differences in population; differences in intervention; surrogate markers; network meta-analysis (0 P)
   2. No important differences in population or intervention; hard clinical outcome (1 P)
5. Funding bias **(1 P)**
   1. Industry funding OR conflict of interest (0 P)
   2. Private institutions, foundations, non-governmental organizations (0.5 P)
   3. Academic institutions, research institutions (1 P)

**2**

1. Study design **(+ 2 P)**

**Overall Score**^3^

95% CI: confidence intervals; P: point(s); RCT: randomized controlled trial.

^1^ ≥2/3 of studies low risk of bias = 0.5 P; >1/3 of studies high risk of bias OR not assessed = 0 P; unclear risk of bias = 0.25P)

^2^ ≥2/3 of overall score = 2 P; ≥1/3 of overall score = 1 P; otherwise = 0 P

^3^ 0-3.49: very low evidence; 3.5-5.49: low evidence; 5.5-6.99: moderate evidence; ≥7: high evidence

NutriGrade scoring system for SRs without MA of cohort studies

1. Risk of bias/ study quality/ study limitations **(2 P)**
   1. No information available (0 P)
   2. Risk of bias (2 P)
      1. Ascertainment of exposure^1^
      2. Adjusted basic & outcome relevant model^1^
      3. Assessment of outcome^1^
      4. Adequacy of follow-up duration^1^
   3. Study quality (2 P)^2^
2. Precision **(1 P)**
   1. <500 Events or <2000 participants events (0 P)
   2. ≥500 Events or ≥2000 participants events (1 P)
3. Heterogeneity **(1 P)**
   1. >1/3 of included studies have an inconsistent result (i.e. point estimates and/or 95% CI did not overlap between studies) (0 P)
   2. ≥2/3 of included studies have a consistent result (i.e. point estimates and/or 95% CI did overlap between studies) (1 P)
4. Directness **(1 P)**
   1. Differences in population; differences in intervention; surrogate markers; network meta-analysis (0 P)
   2. No important differences in population or intervention; hard clinical outcome (1 P)
5. Funding bias **(1 P)**
   1. Industry funding OR conflict of interest (0 P)
   2. Private institutions, foundations, non-governmental organizations (0.5 P)
   3. Academic institutions, research institutions (1 P)
6. Effect size **(2 P)**
   1. No effect for >1/3 of included studies (0 P)
   2. Moderate effect size for ≥2/3 of included studies (1 P)
   3. Large effect size for ≥2/3 of included studies (2 P)

**Overall Score**^3^

95% CI: confidence intervals; P: point(s); RR: risk ratio.

^1^ ≥2/3 of studies low risk of bias = 0.5 P; >1/3 of studies high risk of bias OR not assessed = 0 P; unclear risk of bias = 0.25 P)

^2^ cut-off for different quality scale (≥3/4 of overall score= 2 P; ≥1/2 of overall score= 1 P; <1/2 of overall score= 0 P); i.e. **Newcastle-Ottawa Scale** (mean): ≥7= 2 P; 4-6.9= 1 P; 0-3.9= 0 P;

^3^ 0-2.99: very low evidence; 3-4.49: low evidence; 4.5-5.99: moderate evidence; ≥6: high evidence
