# Supplementary material for: Protein intake and cancer: an umbrella review of systematic reviews for the evidence-based guideline of the German Nutrition Society
Source: Eur J Nutr. 2024 Apr 21;63(5):1471–86. doi: 10.1007/s00394-024-03380-4 (PMC11329548; doi:10.1007/s00394-024-03380-4)
Supplement: Supplementary file 6 — Supplementary file6 (DOCX 28 KB) [file 394_2024_3380_MOESM6_ESM.docx]

**Supplementary Material S6.** List of excluded studies.

| **Reference** | **Reason for exclusion** |
| --- | --- |
| (2022) 2022 Annual Meeting of The North American Menopause Society October 12 - 15, 2022, Atlanta, GA. Menopause 29. | Only abstract available |
| Abid Z, Cross AJ, Sinha R (2014) Meat, dairy, and cancer. Am J Clin Nutr 100, Suppl:386–393. | Irrelevant study type |
| Acham M, Wesselius A, van Osch FHM et al. (2020) Intake of milk and other dairy products and the risk of bladder cancer: a pooled analysis of 13 cohort studies. Eur J Clin Nutr 74:28–35. | Relevant diet-disease relationship not investigated |
| Aiello P, Sharghi M, Mansourkhani SM et al. (2019) Medicinal plants in the prevention and treatment of colon cancer. Oxid Med Cell Longev 2019:2075614. | Irrelevant study type |
| Alegria-Lertxundi I, Bujanda L, Arroyo-Izaga M (2022) Role of dairy foods, fish, white meat, and eggs in the prevention of colorectal cancer: a systematic review of observational studies in 2018-2022. | Relevant diet-disease relationship not investigated |
| Applegate CC, Rowles JL, Ranard KM et al. (2018) Soy consumption and the risk of prostate cancer: an updated systematic review and meta-analysis. FASEB J 2017;31:S1 | Only abstract available |
| Arafa A, Eshak ES, Dong J-Y et al. (2022) Dairy intake and the risk of pancreatic cancer: the Japan Collaborative Cohort Study (JACC Study) and meta-analysis of prospective cohort studies. Br J Nutr 128:1147–1155. | Relevant diet-disease relationship not investigated |
| Arafat HM, Omar J, Shafii N et al. (2023) The association between breast cancer and consumption of dairy products: a systematic review. Ann Med 55:2198256. | Irrelevant exposure |
| Ashari LS, Abd Rashid AA, Mohd Razif S et al. (2023) Diet is Linked to Colorectal Cancer Risk among Asian Adults: A Scoping Review. Malays J Med Sci 30:8–31. | Irrelevant study type |
| Aune D, Lau R, Chan DSM et al. (2012) Dairy products and colorectal cancer risk: a systematic review and meta-analysis of cohort studies. Ann Oncol 23:37–45. | Relevant diet-disease relationship not investigated |
| Aune D, Navarro Rosenblatt, D. A., Chan DS et al. (2015) Dairy products, calcium, and prostate cancer risk: a systematic review and meta-analysis of cohort studies. Am J Clin Nutr 101:87–117. | Relevant diet-disease relationship not investigated |
| Bakken T, Braaten T, Olsen A et al. (2018) Milk and risk of colorectal, colon and rectal cancer in the Norwegian Women and Cancer (NOWAC) cohort study. Br J Nutr 119:1274–1285. | Irrelevant study type |
| Balali A, Askari G, Anjom-Shoae J et al. (2023) Association between nut consumption and prostate cancer risk in adults: A systematic review and dose-response meta-analysis of observational studies. Nutr Metab Cardiovasc Dis 33:1293–1307. | Irrelevant exposure |
| Barrubés L, Babio N, Becerra-Tomás N et al. (2019) Association between dairy product consumption and colorectal cancer risk in adults: a systematic review and meta-analysis of epidemiologic studies. Adv Nutr 10, Suppl 2:S190-S211. | Relevant diet-disease relationship not investigated |
| Bermejo LM, López-Plaza B, Santurino C et al. (2019) Milk and dairy product consumption and bladder cancer risk: a systematic review and meta-analysis of observational studies. Adv Nutr 10, Suppl 2:S224-S238. | Relevant diet-disease relationship not investigated |
| Bloomfield HE, Kane R, Koeller E et al. (2015) Benefits and harms of the mediterranean diet compared to other diets, Washington (DC) | Irrelevant exposure |
| Body J-J (2015) Dairy products: facts and fiction: World Congress on osteoporosis, osteoarthritis and musculoskeletal diseases (WCO-IOF-ESCEO 2015): satellite symposia abstracts. Osteoporos Int 26, Suppl 1:383. | Only abstract available |
| Boushey C, Ard J, Bazzano L et al. (2020) Dietary patterns and breast, colorectal, lung, and prostate cancer: a systematic review. | Relevant diet-disease relationship not investigated |
| Caini S, Masala G, Gnagnarella P et al. (2016) Food of animal origin and risk of non-Hodgkin lymphoma and multiple myeloma: a review of the literature and meta-analysis. Crit Rev Oncol Hematol 100:16–24. | Relevant diet-disease relationship not investigated |
| Cao C, Gan X, He Y et al. (2023) Association between nut consumption and cancer risk: a meta-analysis. | Irrelevant exposure |
| Carpio K, Cornejo V, Leal-Witt MJ et al. (2021) Consumo de lácteos y riesgo de cáncer colorrectal: Una revisión de la literatura científica. Rev chil nutr 48:405–413. | Irrelevant language |
| Chen L, Li M, Li H (2019) Milk and yogurt intake and breast cancer risk: a meta-analysis. Medicine (Baltimore) 98:e14900. | Relevant diet-disease relationship not investigated |
| Chidike Ezeorba TP, Ezugwu AL, Chukwuma IF et al. (2024) Health-promoting properties of bioactive proteins and peptides of garlic (Allium sativum). Food Chem 435:137632. | Irrelevant outcome |
| Connolly G, Clark CM, Campbell RE et al. (2022) Poultry consumption and human health: how much is really known? a systematically searched scoping review and research perspective. Adv Nutr 13:2115–2124. | Relevant diet-disease relationship not investigated |
| Contreras García E, Zaragoza-Martí A (2020) Influencia de la ingesta de alimentos o grupos de alimentos en la aparición y/o protección de los diversos tipos de cáncer: Revisión sistemática (Influence of food or food groups intake on the occurrence and/or protection of different types of cancer: systematic review). Nutr Hosp 37:169–192. | Irrelevant language |
| de Waure C, Quaranta G, Gualano MR et al. (2015) Systematic review of studies investigating the association between dietary habits and cutaneous malignant melanoma. Public Health 129:1099–1113. | Irrelevant exposure |
| Dong JY, Zhang L, He K et al. (2011) Dairy consumption and risk of breast cancer: a meta-analysis of prospective cohort studies. Breast Cancer Res Treat 127:23–31. | Relevant diet-disease relationship not investigated |
| El Kinany K, Deoula M, Hatime Z et al. (2018) Dairy products and colorectal cancer in middle eastern and north African countries: a systematic review. BMC Cancer 18:233. | Relevant diet-disease relationship not investigated |
| Fardet A, Boirie Y (2014) Associations between food and beverage groups and major diet-related chronic diseases: an exhaustive review of pooled/meta-analyses and systematic reviews. Nut Rev 72:741–762. | Irrelevant study type |
| Gao X, Jia H-Y, Chen G-C et al. (2020) Yogurt intake reduces all-cause and cardiovascular disease mortality: a meta-analysis of eight prospective cohort studies. Chin J Integr Med 26:462–468. | Irrelevant outcome |
| García EV, Sala-Serra M, Continente-Garcia X et al. (2020) The association between breast cancer and consumption of dairy products: a systematic review (The association between breast cancer and consumption of dairy products: a systematic review). Nutr Hosp 34:589–598. | Relevant diet-disease relationship not investigated |
| Gathirua-Mwangi WG, Zhang J (2014) Dietary factors and risk for advanced prostate cancer. Eur J Cancer Prev 23:96–109. | Critically low AMSTAR 2 rating |
| Genkinger JM, Wang M, Li R et al. (2014) Dairy products and pancreatic cancer risk: a pooled analysis of 14 cohort studies. Ann Oncol 25:1106–1115. | Relevant diet-disease relationship not investigated |
| George ES, Sood S, Broughton A et al. (2021) The association between diet and hepatocellular carcinoma: a systematic review. Nutrients 13:172. | Relevant diet-disease relationship not investigated |
| Ghaffari HR, Yunesian M, Nabizadeh R et al. (2019) Environmental etiology of gastric cancer in Iran: a systematic review focusing on drinking water, soil, food, radiation, and geographical conditions. Environ Sci Pollut Res Int 26:10487–10495. | Relevant diet-disease relationship not investigated |
| Gil H, Chen Q-Y, Khil J et al. (2022) Milk intake in early life and later cancer risk: a meta-analysis. Nutrients 14:1233. | Relevant diet-disease relationship not investigated |
| Gille D, Schmid A, Walther B et al. (2018) Fermented food and non-communicable chronic diseases: a review. Nutrients 10. | Irrelevant study type |
| Godos J, Bella F, Sciacca S et al. (2017) Vegetarianism and breast, colorectal and prostate cancer risk: an overview and meta-analysis of cohort studies. J Hum Nutr Diet 30:349–359. | Irrelevant exposure |
| Guo L-L, Li Y-T, Yao J et al. (2021) Dairy consumption and risk of conventional and serrated precursors of colorectal cancer: a systematic review and meta-analysis of observational studies. J Oncol 2021:9948814. | Relevant diet-disease relationship not investigated |
| Guo Y, Shan Z, Ren H et al. (2015) Dairy consumption and gastric cancer risk: a meta-analysis of epidemiological studies. Nutr Cancer 67:555–568. | Relevant diet-disease relationship not investigated |
| Gupta M, Asfaha DM, Ponnaiah G (2023) Millets: A Nutritional Powerhouse With Anti-cancer Potential. Cureus 15:e47769. | Irrelevant outcome |
| Harrison S, Lennon R, Holly J et al. (2017) Does milk intake promote prostate cancer initiation or progression via effects on insulin-like growth factors (IGFs)? A systematic review and meta-analysis. Cancer Causes Control 28:497–528. | Irrelevant outcome |
| He Y, Tao Q, Zhou F et al. (2021) The relationship between dairy products intake and breast cancer incidence: a meta-analysis of observational studies. BMC Cancer 21:1109. | Relevant diet-disease relationship not investigated |
| Huber J, Imhof M, Schmidt M (2010) Effects of soy protein and isoflavones on circulating hormone concentrations in pre- and post-menopausal women: a systematic review and meta-analysis. Hum Reprod Update 16:110-111. | Irrelevant study type |
| ~~Huncharek M, Muscat J, Kupelnick B (2009) Colorectal cancer risk and dietary intake~~  ~~of calcium, vitamin d, and dairy products: a meta-analysis of 26,335 cases from 60 observational studies. Nutr Cancer 61:47–69~~ | ~~Other~~ |
| Hwang YW, Kim SY, Jee SH et al. (2009) Soy food consumption and risk of prostate cancer: a meta-analysis of observational studies. Nutr Cancer 61:598–606 | Relevant diet-disease relationship not investigated |
| Ibrahim MO, Abuhijleh H, Tayyem R (2023) What Dietary Patterns and Nutrients are Associated with Pancreatic Cancer? Literature Review. Cancer management and research 15:17–30. | Irrelevant study type |
| Jamioł-Milc D, Biernawska J, Liput M et al. (2021) Seafood intake as a method of non-communicable diseases (NCD) prevention in adults. Nutrients 13:1422. https://doi.org/10.3390/nu13051422 | Relevant diet-disease relationship not investigated |
| Jeyaraman MM, Abou-Setta AM, Grant L et al. (2019) Dairy product consumption and development of cancer: an overview of reviews. BMJ Open 9:e023625. https://doi.org/10.1136/bmjopen-2018-023625 | Irrelevant study type |
| Jin S, Je Y (2022) Dairy Consumption and Total Cancer and Cancer-Specific Mortality: A Meta-Analysis of Prospective Cohort Studies. Adv Nutr 13:1063–1082. | Relevant diet-disease relationship not investigated |
| Jin S, Kim Y, Je Y (2020) Dairy consumption and risks of colorectal cancer incidence and mortality: a meta-analysis of prospective cohort studies. Cancer Epidemiol Biomarkers Prev 29:2309–2322. | Relevant diet-disease relationship not investigated |
| Kado K, Forsyth A, Patel PR et al. (2012) Dietary supplements and natural products in breast cancer trials. Front Biosci (Elite Ed) 4:546–567 | Irrelevant exposure |
| Kang JH, Dong Z, Shin SH (2023) Benefits of Soybean in the Era of Precision Medicine: A Review of Clinical Evidence. Journal of microbiology and biotechnology 33:1552–1562. | Irrelevant study type |
| Kapsetaki SE, Marquez Alcaraz G, Maley CC et al. (2022) Diet, microbes, and cancer across the tree of life: a systematic review. Curr Nutr Rep 11:508–525. | Relevant diet-disease relationship not investigated |
| Kazemi A, Barati-Boldaji R, Soltani S et al. (2021) Intake of various food groups and risk of breast cancer: a systematic review and dose-response meta-analysis of prospective studies. Adv Nutr 12:809–849. | Relevant diet-disease relationship not investigated |
| Khankari NK, Yang JJ, Sawada N et al. (2020) Soy intake and colorectal cancer risk: results from a pooled analysis of prospective cohort studies conducted in China and Japan. J Nutr 150:2442–2450. | Irrelevant study type |
| Kim CE, Yoon LS, Michels KB et al. (2022) The Impact of Prebiotic, Probiotic, and Synbiotic Supplements and Yogurt Consumption on the Risk of Colorectal Neoplasia among Adults: A Systematic Review. Nutrients 14. | Irrelevant exposure |
| Kong F, Geng E, Ning J et al. (2020) The association between dietary protein intake and esophageal cancer risk: a meta-analysis. Biosci Rep 40:BSR20193692. | Irrelevant study type |
| Kongerslev Thorning T, Raben A, Tholstrup T et al. (2016) Milk and dairy products: good or bad for human health? An assessment of the totality of scientific evidence. Food Nutr Res 60:32527 | Irrelevant study type |
| Lampe JW (2011) Dairy products and cancer. J Am Coll Nutr 30, Suppl 1:464S–470S. | Irrelevant study type |
| Larsson SC, Crippa A, Orsini N et al. (2015) Milk consumption and mortality from all causes, cardiovascular disease, and cancer: a systematic review and meta-analysis. | Relevant diet-disease relationship not investigated |
| Latino-Martel P, Cottet V, Druesne-Pecollo N et al. (2016) Alcoholic beverages, obesity, physical activity and other nutritional factors, and cancer risk: A review of the evidence. Crit Rev Oncol Hematol 99:308–323. | Relevant diet-disease relationship not investigated |
| Li F, An S, Zhou Y et al. (2011) Milk and dairy consumption and risk of bladder cancer: a meta-analysis. Urology 78:1298–1305. | Relevant diet-disease relationship not investigated |
| Li B-L, Jiang G-X, Xue Q et al. (2016) Dairy consumption and risk of esophageal squamous cell carcinoma: a meta-analysis of observational studies. Asia Pac J Clin Oncol 12:e269-e279. | Relevant diet-disease relationship not investigated |
| Li X, Wu L, Chen H et al. (2019) P2.16-39 The Association Between Dietary Protein Intake and the Risk of Lung Cancer: A Meta-Analysis. Journal of Thoracic Oncology 14:S881. | Irrelevant study type |
| Li X, Zhao J, Li P et al. (2018) Dairy products intake and endometrial cancer risk: a meta-analysis of observational studies. Nutrients 10. | Relevant diet-disease relationship not investigated |
| Liang Z, Song X, Hu J et al. (2022) Fermented dairy food intake and risk of colorectal cancer: a systematic review and meta-analysis. Front Oncol 12:812679. | Relevant diet-disease relationship not investigated |
| Liao M-Q, Gao X-P, Yu X-X et al. (2020) Effects of dairy products, calcium and vitamin D on ovarian cancer risk: a meta-analysis of twenty-nine epidemiological studies. Br J Nutr 124:1001–1012. | Relevant diet-disease relationship not investigated |
| Lionel Lafay, Raphaëlle Ancellin, Lafay L et al. (2015) Alimentation et cancer colorectal. Cahiers de Nutrition et de Diététique 50:262–270. | Irrelevant language |
| Liu J, Tang W, Sang L et al. (2015) Milk, yogurt, and lactose intake and ovarian cancer risk: a meta-analysis. Nutr Cancer 67:68–72. | Relevant diet-disease relationship not investigated |
| López-Plaza B, Bermejo LM, Santurino C et al. (2019) Milk and dairy product consumption and prostate cancer risk and mortality: an overview of systematic reviews and meta-analyses. Adv Nutr 10, Suppl 2:S212-S223. | Irrelevant study type |
| Lu W, Chen H, Niu Y et al. (2016) Dairy products intake and cancer mortality risk: a meta-analysis of 11 population-based cohort studies. Nutr J 15:91. | Relevant diet-disease relationship not investigated |
| Lumsden AL, Mulugeta A, Hyppönen E (2023) Milk consumption and risk of twelve cancers: a large-scale observational and Mendelian randomisation study. Clin Nutr 42:1–8. | Irrelevant study type |
| Ma RW-L, Chapman K (2009) A systematic review of the effect of diet in prostate cancer prevention and treatment. J Hum Nutr Diet 22:187–199. | Relevant diet-disease relationship not investigated |
| Mandair D, Rossi RE, Pericleous M et al. (2014) Prostate cancer and the influence of dietary factors and supplements: a systematic review. Nutr Metab (Lond) 11:30. | Irrelevant exposure |
| Manimurugan C, Sujatha M, Rathnakumar AL et al. (2023) Role of flaxseed (Linum usitatissimum L.) in disease prevention and treatment. Asian Pac J Trop Biomed 13:277. | Irrelevant study type |
| Mao Q-Q, Dai Y, Lin Y-W et al. (2011) Milk consumption and bladder cancer risk: a meta-analysis of published epidemiological studies. Nutr Cancer 63:1263–1271. | Relevant diet-disease relationship not investigated |
| Martini LA, Wood RJ (2009) Milk intake and the risk of type 2 diabetes mellitus, hypertension and prostate cancer. Arq Bras Endocrinol Metabol 53:688–694 | Irrelevant study type |
| Mazidi M, Mikhailidis DP, Howard G et al. (2018) Consumption of dairy product and its association with total and cause specific mortality - a population-based cohort study and meta-analysis. Eur Heart J 39:1883–1948. | Only abstract available |
| Mazidi M, Mikhailidis DP, Sattar N et al. (2018) Consumption of dairy product and its association with total and cause specific mortality - a population-based cohort study and meta-analysis. Clin Nutr 38:2833–2845. | Relevant diet-disease relationship not investigated |
| McEvoy CT, Temple N, Woodside JV (2012) Vegetarian diets, low-meat diets and health: a review. Public Health Nutr 15:2287–2294. | Irrelevant exposure |
| Mena P, Angelino D (2020) Plant food, nutrition, and human health. Nutrients 12:2157. | Irrelevant study type |
| Messina M (2014) Soy foods, isoflavones, and the health of postmenopausal women. Am J Clin Nutr 100, Suppl 1:423S-30S. | Irrelevant study type |
| Mohseni R, Mohseni F, Alizadeh S et al. (2020) The association of dietary approaches to stop hypertension (DASH) diet with the risk of colorectal cancer: a meta-analysis of observational studies. Nutr Cancer 72:778–790. | Irrelevant exposure |
| Myung SK, Ju W, Choi HJ et al. (2009) Soy intake and risk of endocrine-related gynaecological cancer: a meta-analysis. | Relevant diet-disease relationship not investigated |
| Nachvak SM, Moradi S, Anjom-Shoae J et al. (2019) Soy, soy isoflavones, and protein intake in relation to mortality from all causes, cancers, and cardiovascular diseases: a systematic review and dose-response meta-analysis of prospective cohort studies. J Acad Nutr Diet 119:1483-1500.e17. | Irrelevant outcome |
| Naghshi S, Sadeghi O, Willett WC et al. (2020) Dietary intake of total, animal, and plant proteins and risk of all cause, cardiovascular, and cancer mortality: systematic review and dose-response meta-analysis of prospective cohort studies. BMJ 370:m2412. | Irrelevant outcome |
| Naghshi S, Sadeghi O, Larijani B et al. (2021) High vs. low-fat dairy and milk differently affects the risk of all-cause, CVD, and cancer death: a systematic review and dose-response meta-analysis of prospective cohort studies. Crit Rev Food Sci Nutr:1–15. | Irrelevant outcome |
| Naghshi S, Sadeghian M, Nasiri M et al. (2021) Association of total nut, tree nut, peanut, and peanut butter consumption with cancer incidence and mortality: a comprehensive systematic review and dose-response meta-analysis of observational studies. Adv Nutr 12:793–808. | Relevant diet-disease relationship not investigated |
| Nanri A, Mizoue T, Shimazu T et al. (2017) Dietary patterns and all-cause, cancer, and cardiovascular disease mortality in Japanese men and women: the Japan public health center-based prospective study. PLoS One 12:e0174848. | Irrelevant study type |
| Niclis C, Díaz, María del Pilar, Eynard AR et al. (2012) Dietary habits and prostate cancer prevention: a review of observational studies by focusing on South America. Nutr Cancer 64:23–33. | Relevant diet-disease relationship not investigated |
| O'Connor LE, Gifford CL, Woerner DR et al. (2020) Dietary meat categories and descriptions in chronic disease research are substantively different within and between experimental and observational studies: a systematic review and landscape analysis. Adv Nutr 11:41–51. | Irrelevant exposure |
| Oleksiak A, Deptała A, Dąbrowska-Bender M et al. (2022) Intake of selected food groups in relation to risk of breast cancer. Ann Agric Environ Med 29:342–347. | Irrelevant study type |
| Papadimitriou N, Bouras E, van den Brandt PA et al. (2021) A prospective diet-wide association study for risk of colorectal cancer in EPIC. Clin Gastroenterol Hepatol. | Only abstract available |
| Prentice AM (2014) Dairy products in global public health. Am J Clin Nutr 99, Suppl:1212S–1216S. | Relevant diet-disease relationship not investigated |
| Qi X-X, Shen P (2020) Associations of dietary protein intake with all-cause, cardiovascular disease, and cancer mortality: a systematic review and meta-analysis of cohort studies. Nutr Metab Cardiovasc Dis 30:1094–1105. | Irrelevant outcome |
| Qin L-Q, He K, Xu J-Y (2009) Milk consumption and circulating insulin-like growth factor-I level: a systematic literature review. Int J Food Sci Nutr 60, Suppl 7:330–340. | Irrelevant outcome |
| Quach P, El Sherif R, Gomes J et al. (2017) A systematic review of the risk factors associated with the onset and progression of primary brain tumours. Neurotoxicology 61:214–232. | Irrelevant exposure |
| Ralston RA, Truby H, Palermo CE et al. (2014) Colorectal cancer and nonfermented milk, solid cheese, and fermented milk consumption: a systematic review and meta-analysis of prospective studies. Crit Rev Food Sci Nutr 54:1167–1179. | Relevant diet-disease relationship not investigated |
| Rodriguez-Archilla A, Gomez-Fernandez M (2023) Influence of dairy products consumption on oral cancer risk: A meta-analysis. J Dent Res Dent Clin Dent Prospects 17:1–7. | Relevant diet-disease relationship not investigated |
| Rohrmann S, Hermann S (2021) Onkologische Prävention – inwiefern ist die Ernährung entscheidend? Onkologe 27:100–107. | Irrelevant study type |
| Rohrmann S, van Hemelrijck M (2015) The association of milk and dairy consumption and calcium intake with the risk and severity of prostate cancer. Curr Nutr Rep 4:66–71. | Irrelevant study type |
| Rundle-Thiele D, Shrestha S, Janda M (2022) Prevention of endometrial cancer through lifestyle Interventions: a systematic review and synthesis. Gynecol Oncol Rep 39:100900. | Irrelevant exposure |
| Samanta SK, Choudhury P, Sarma PP et al. (2022) Dietary phytochemicals/nutrients as promising protector of breast cancer development: a comprehensive analysis. Pharmacol Rep 74:583–601. | Irrelevant study type |
| Santarelli RL, Pierre F, Corpet DE (2008) Processed meat and colorectal cancer: a review of epidemiologic and experimental evidence. Nutr Cancer 60:131–144. | Irrelevant language |
| Sargsyan A, Dubasi HB (2021) Milk consumption and prostate cancer: a systematic review. World J Mens Health 39:419–428. | Relevant diet-disease relationship not investigated |
| Savaiano DA, Hutkins RW (2021) Yogurt, cultured fermented milk, and health: a systematic review. Nut Rev 79:599–614. | Relevant diet-disease relationship not investigated |
| Schwingshackl L, Boeing H, Stelmach-Mardas M et al. (2017) Dietary supplements and risk of cause-specific death, cardiovascular disease, and cancer: a systematic review and meta-analysis of primary prevention trials. Adv Nutr 8:27–39. | Irrelevant exposure |
| Schwingshackl L, Knüppel S, Michels N et al. (2019) Intake of 12 food groups and disability-adjusted life years from coronary heart disease, stroke, type 2 diabetes, and colorectal cancer in 16 European countries. Eur J Epidemiol 34:765–775. | Relevant diet-disease relationship not investigated |
| Schwingshackl L, Schwedhelm C, Hoffmann G et al. (2018) Food groups and risk of colorectal cancer. Int J Cancer 142:1748–1758. | Relevant diet-disease relationship not investigated |
| Sehrawat N, Yadav M, Kumar S et al. (2023) Mung bean as a potent emerging functional food having anticancer therapeutic potential: mechanistic insight and recent updates. Biotechnol Appl Biochem 70:2002–2016. | Irrelevant study type |
| Sergentanis TN, Ntanasis-Stathopoulos I, Tzanninis I-G et al. (2019) Meat, fish, dairy products and risk of hematological malignancies in adults - a systematic review and meta-analysis of prospective studies. Leuk Lymphoma 60:1978–1990. | Relevant diet-disease relationship not investigated |
| Shin J, Millstine D, Ruddy B et al. (2019) Effect of plant- and animal-based foods on prostate cancer risk. J Am Osteopath Assoc. | Relevant diet-disease relationship not investigated |
| Shu L, Wang X-Q, Wang S-F et al. (2013) Dietary patterns and stomach cancer: a meta-analysis. Nutr Cancer 65:1105–1115. | Irrelevant exposure |
| Signal V, Huang S, Sarfati D et al. (2018) Dairy consumption and risk of testicular cancer: a systematic review. Nutr Cancer 70:710–736. | Relevant diet-disease relationship not investigated |
| Sun Y, Lin L-J, Sang L-X et al. (2014) Dairy product consumption and gastric cancer risk: a meta-analysis. World J Gastroenterol 20:15879–15898. | Relevant diet-disease relationship not investigated |
| Tabung FK, Brown LS, Fung TT (2017) Dietary patterns and colorectal cancer risk: a review of 17 years of evidence (2000-2016). Curr Colorectal Cancer Rep 13:440–454. | Irrelevant exposure |
| Tan ST, Tan SS, Tan CX (2023) Soy protein, bioactive peptides, and isoflavones: A review of their safety and health benefits. PharmaNutrition 25:100352. | Irrelevant exposure |
| Tanitame M, Sugawara Y, Lu Y et al. (2023) Dairy consumption and incident risk of thyroid cancer in Japan: a pooled analysis of the Miyagi Cohort Study and the Ohsaki Cohort Study. Eur J Nutr 62:251–259. | Irrelevant study type |
| Tian S, Yu J, Kang W et al. (2014) Association between dairy intake and gastric cancer: a meta-analysis of observational studies. PLoS One 9:e101728. | Relevant diet-disease relationship not investigated |
| Travis RC, Appleby PN, Siddiq A et al. (2013) Genetic variation in the lactase gene, dairy product intake and risk for prostate cancer in the European prospective investigation into cancer and nutrition. Int J Cancer 132:1901–1910. | Irrelevant study type |
| Urzì AG, Tropea E, Gattuso G et al. (2023) Ketogenic Diet and Breast Cancer: Recent Findings and Therapeutic Approaches. Nutrients 15:4357. | Relevant diet-disease relationship not investigated |
| van Die, M Diana, Bone KM, Williams SG et al. (2014) Soy and soy isoflavones in prostate cancer: a systematic review and meta-analysis of randomized controlled trials. BJU Int 113:30. | Irrelevant exposure |
| Vargas AJ, Thompson PA (2012) Diet and nutrient factors in colorectal cancer risk. Nutr Clin Pract 27:613–623. | Irrelevant study type |
| Vasconcelos A, Santos T, Ravasco P et al. (2019) Dairy products: is there an impact on promotion of prostate cancer? a review of the literature. Front Nutr 6:62. | Relevant diet-disease relationship not investigated |
| Ventriglio A, Sancassiani F, Contu MP et al. (2020) Mediterranean diet and its benefits on health and mental health: a literature review. Clin Pract Epidemiol Ment Health 16, Suppl 1:156–164. | Irrelevant exposure |
| Vieira AR, Abar L, Chan DSM et al. (2017) Foods and beverages and colorectal cancer risk: a systematic review and meta-analysis of cohort studies, an update of the evidence of the WCRF-AICR Continuous Update Project. Ann Oncol 28:1788–1802. | Relevant diet-disease relationship not investigated |
| Wal A, Singh MR, Gupta A et al. (2024) Pumpkin seeds (Cucurbita spp.) as a nutraceutical used in various lifestyle disorders. NPJ 14. | Irrelevant exposure |
| Wang J, Li X, Zhang D (2016) Dairy product consumption and risk of non-hodgkin lymphoma: a meta-analysis. Nutrients 8:120. | Relevant diet-disease relationship not investigated |
| Wang S, Zhou M, Ji A et al. (2018) Milk/dairy products consumption and gastric cancer: an update meta-analysis of epidemiological studies. Oncotarget 9:7126–7135. | Relevant diet-disease relationship not investigated |
| WCRF (World Cancer Research Fund), AICR (American Institute for Cancer Research) (2015) The associations between diet, nutrition and physical activity and the risk of kidney cancer. Continuous update project. | Critically low AMSTAR 2 rating |
| Wei Y, Lv J, Guo Y et al. (2020) Soy intake and breast cancer risk: aprospective study of 300,000 Chinese women and a dose-response meta-analysis. Eur J Epidemiol 35:567–578. | Irrelevant study type |
| Wiggs AG, Chandler JK, Aktas A et al. (2021) The effects of diet and exercise on endogenous estrogens and subsequent breast cancer risk in postmenopausal women. Front Endocrinol (Lausanne) 12:732255. | Irrelevant exposure |
| Wu Y, Huang R, Wang M et al. (2021) Dairy foods, calcium, and risk of breast cancer overall and for subtypes defined by estrogen receptor status: a pooled analysis of 21 cohort studies. Am J Clin Nutr 114:450–461. | Relevant diet-disease relationship not investigated |
| Wu SH, Liu Z (2013) Soy food consumption and lung cancer risk: a meta-analysis using a common measure across studies. Nutr Cancer 65:625–632. | Critically low AMSTAR 2 rating |
| Wu J, Yu Y, Huang L et al. (2020) Dairy product consumption and bladder cancer risk: a meta-analysis. Nutr Cancer 72:377–385. | Relevant diet-disease relationship not investigated |
| Wu J, Zeng R, Huang J et al. (2016) Dietary protein sources and incidence of breast cancer: a dose-response meta-analysis of prospective studies. Nutrients 8. | Relevant diet-disease relationship not investigated |
| Xiao J, Ma J, Khan MZ et al. (2023) Unlocking the potential of milk whey protein components in colorectal cancer prevention and therapy. Crit Rev Food Sci Nutr:1–38. | Irrelevant study type |
| Yang W-S, Va P, Wong M-Y et al. (2011) Soy intake is associated with lower lung cancer risk: results from a meta-analysis of epidemiologic studies. Am J Clin Nutr 94:1575–1583. | Relevant diet-disease relationship not investigated |
| Yang Y, Wang X, Yao Q et al. (2016) Dairy product, calcium intake and lung cancer risk: a systematic review with meta-analysis. Sci Rep 6:20624. | Relevant diet-disease relationship not investigated |
| Yang Y, Zhou J, Yang Y et al. (2017) Systematic review and meta-analysis: dairy consumption and hepatocellular carcinoma risk. J Public Health 25:591–599. | Relevant diet-disease relationship not investigated |
| Yan L, Spitznagel EL: Soy, isoflavones and prostate cancer risk in men: a meta-analysis. FASEB J 2009;23:S1. | Only abstract available |
| Yu Y, Li H, Xu K et al. (2016) Dairy consumption and lung cancer risk: a meta-analysis of prospective cohort studies. Onco Targets Ther 9:111–116. | Relevant diet-disease relationship not investigated |
| Yuan J, Li W, Sun W et al. (2019) Milk and dairy products consumption and the risk of oral or oropharyngeal cancer: a meta-analysis. Biosci Rep 39:BSR20193526. | Relevant diet-disease relationship not investigated |
| Zang J, Shen M, Du S et al. (2015) The association between dairy intake and breast cancer in Western and Asian populations: a systematic review and meta-analysis. J Breast Cancer 18:313–322. | Relevant diet-disease relationship not investigated |
| Zhang FF, Cudhea F, Shan Z et al. (2019) Preventable cancer burden associated with poor diet in the United States. JNCI cancer spectrum 3:pkz034. | Irrelevant study type |
| Zhang K, Dai H, Liang W et al. (2019) Fermented dairy foods intake and risk of cancer. Int J Cancer 144:2099–2108. | Relevant diet-disease relationship not investigated |
| Zhang Z-H, Yang L-S, Sun Y-H (2012) Re: Li et al.: Milk and dairy consumption and risk of bladder cancer: a meta-analysis (Urology 2011;78:1298-1305). Urology 80:231-232. | Irrelevant study type |
| Zhang Z-H, Yang L-S, Sun Y-H (2012) The association between milk consumption and bladder cancer risk: appraisal of a recent meta-analysis. Nutr Cancer 64:1288–1289. | Irrelevant study type |
| Zhao Q, He Y, Wang K et al. (2021) Dairy consumption and liver cancer risk: a systematic review and dose-response meta-analysis of observational studies. Nutr Cancer 73:1–17. | Only abstract available |
| Zhao T-T, Jin F, Li J-G et al. (2019) Dietary isoflavones or isoflavone-rich food intake and breast cancer risk: a meta-analysis of prospective cohort studies. Clin Nutr 38:136–145. | Irrelevant exposure |
| Zhao Z, Wu D, Gao S et al. (2023) The association between dairy products consumption and prostate cancer risk: a systematic review and meta-analysis. Br J Nutr 129:1714–1731. | Relevant diet-disease relationship not investigated |
| Zhong X, Zhang C (2012) Soy food intake and breast cancer risk: a meta-analysis. Wei Sheng Yan Jiu 41:670–676 | Irrelevant language |
