# Supplementary material for: Protein intake and cancer: an umbrella review of systematic reviews for the evidence-based guideline of the German Nutrition Society
Source: Eur J Nutr. 2024 Apr 21;63(5):1471–86. doi: 10.1007/s00394-024-03380-4 (PMC11329548; doi:10.1007/s00394-024-03380-4)
Supplement: Supplementary file 7 — Supplementary file7 (DOCX 21 KB) [file 394_2024_3380_MOESM7_ESM.docx]

Supplementary Material S7. Methodological quality assessment of systematic reviews using AMSTAR 2.

| **Assessment**  **item**  **Systematic review** | 1. Components of PICO included? | 1. Method a priori? Protocol reported? | 1. Comprehensive literature search strategy? | 1. Study selection performed in duplicate? | 1. Data extraction performed in duplicate? | 1. Number of excluded studies and corresponding reasons provided? | 1. Detailed study characteristics provided? | 1. Risk of bias assessed? | 1. Statistical heterogeneity assessed? | 1. Risk of bias considered in the discussion and interpretation? | 1. Discussion of any heterogeneity observed in the results? | 1. Publication bias investigated? | 1. Likely impact of publication bias discussed? | 1. Potential conflicts of interest stated? | Number of critical weaknesses | Number of non-critical weaknesses | **Methodological quality** |
| --- | --- | --- | --- | --- | --- | --- | --- | --- | --- | --- | --- | --- | --- | --- | --- | --- | --- |
| Shin 2023 [13] | Yes | Yes | Yes | Yes | Yes | Yes | No | Yes | Yes | No | No | Yes | Yes | Yes | 0 | 3 | **Moderate** |
| Alzahrani 2022 [14] | Yes | Yes | Yes | No | No | Yes | No | Yes | Yes | No | Yes | Yes | Yes | Yes | 0 | 4 | **Moderate** |
| Fan 2022 [15] | Yes | Yes | Yes | Yes | Yes | Yes | No | Yes | Yes | No | No | Yes | No | Yes | 0 | 4 | **Moderate** |
| Zhang 2022 [16] | No | No | Yes | No | Yes | Yes | No | No | Yes | No | Yes | Yes | Yes | No | 1 | 6 | **Low** |
| Ubago-Guisado 2021 [17] | Yes | Yes | Yes | Yes | Yes | Yes | No | Yes | No MA | No | No MA | No MA | No MA | Yes | 0 | 2 | **Moderate** |
| Khodavandi 2020 [18] | No | Yes | Yes | No | Yes | Yes | Yes | Yes | Yes | No | No | No | No | Yes | 1 | 5 | **Low** |
| Mao 2018 [19] | Yes | No | Yes | No | Yes | Yes | Yes | No | Yes | No | Yes | Yes | Yes | Yes | 1 | 3 | **Low** |
| Pang 2018 [20] | Yes | No | Yes | Yes | Yes | Yes | Yes | No | Yes | Yes | Yes | Yes | Yes | Yes | 1 | 1 | **Low** |
| Lai 2017 [21] | Yes | Yes | Yes | No | Yes | Yes | Yes | Yes | Yes | Yes | Yes | Yes | Yes | Yes | 1 | 2 | **High** |
| Pedersen 2013 [22] | Yes | Yes | Yes | Yes | No | Yes | Yes | Yes | No MA | No | No MA | No MA | No MA | Yes | 0 | 3 | **Moderate** |
| Gathirua-Mwangi 2013 [26] | Yes | No | No | No | No | Yes | Yes | No | No MA | No | No MA | No MA | No MA | Yes | 2 | 4 | **Critically low** |
| WCRF 2015 [27] | Yes | Yes | No | Yes | Yes | Yes | Yes | No | Yes | No | No | No | No | Yes | 3 | 3 | **Critically low** |
| Wu 2013 [28] | Yes | No | No | No | Yes | Yes | Yes | No | Yes | No | No | Yes | Yes | Yes | 2 | 4 | **Critically low** |

Abbreviations: AMSTAR 2: A Measurement Tool to Assess Systematic Reviews 2; MA: meta-analysis

The assessment items are provided as shortened versions. The full questionnaire is provided in Supplementary Materials (S3). Critical assessment items are underlined.

High rating = no critical weakness with no or one non-critical weakness: the systematic review provides an accurate and comprehensive summary of the results of the available studies that address the question of interest. Moderate rating = no critical weakness with more than one non-critical weakness: the systematic review has more than one weakness but no critical flaws. It may provide an accurate summary of the results of the available studies that were included in the review. Low rating = one critical weakness with or without non-critical weaknesses: the review has a critical flaw and may not provide an accurate and comprehensive summary of the available studies that address the question of interest. Critically low rating = more than one critical weakness with or without non-critical weaknesses: the review has more than one critical flaw and should not be relied on to provide an accurate and comprehensive summary of the available studies. Shea BJ, Reeves BC, Wells G, Thuku M, Hamel C, Moran J, et al. AMSTAR 2: a critical appraisal tool for systematic reviews that include randomised or non-randomised studies of healthcare interventions, or both. BMJ. 2017 Sep;358(j4008):1-8.
